# Overview of the hypnodensity approach to scoring sleep for polysomnography and home sleep testing

**DOI:** 10.3389/frsle.2023.1163477

**Published:** 2023-04-17

**Authors:** Peter Anderer, Marco Ross, Andreas Cerny, Ray Vasko, Edmund Shaw, Pedro Fonseca

**Affiliations:** ^1^Philips Sleep and Respiratory Care, Vienna, Austria; ^2^The Siesta Group Schlafanalyse GmbH, Vienna, Austria; ^3^Philips Sleep and Respiratory Care, Pittsburgh, PA, United States; ^4^Philips Research, Eindhoven, Netherlands; ^5^Department of Electrical Engineering, Eindhoven University of Technology, Eindhoven, Netherlands

**Keywords:** hypnogram, hypnodensity, sleep stage ambiguity, sleep stage continuity, machine learning, cardiorespiratory sleep staging, hypoxic burden

## Abstract

Human experts scoring sleep according to the American Academy of Sleep Medicine (AASM) rules are forced to select, for every 30-second epoch, one out of five stages, even if the characteristics of the neurological signals are ambiguous, a very common occurrence in clinical studies. Moreover, experts cannot score sleep in studies where these signals have not been recorded, such as in home sleep apnea testing (HSAT). In this topic review we describe how artificial intelligence can provide consistent and reliable scoring of sleep stages based on neurological signals recorded in polysomnography (PSG) and on cardiorespiratory signals recorded in HSAT. We also show how estimates of sleep stage probabilities, usually displayed as hypnodensity graph, can be used to quantify sleep stage ambiguity and stability. As an example of the application of hypnodensity in the characterization of sleep disordered breathing (SDB), we compared 49 patients with sleep apnea to healthy controls and revealed a severity-depending increase in ambiguity and decrease in stability during non-rapid eye movement (NREM) sleep. Moreover, using autoscoring of cardiorespiratory signals, we show how HSAT-derived apnea-hypopnea index and hypoxic burden are well correlated with the PSG indices in 80 patients, showing how using this technology can truly enable HSATs as alternatives to PSG to diagnose SDB.

## Introduction

Human sleep stage scoring was developed to summarize the information of electroencephalogram (EEG), electrooculogram (EOG) and electromyogram (EMG) correlates of normal sleep for healthy subjects (Rechtschaffen and Kales, 1968). These neurological signals provide the basic information requisite for visually differentiating sleep stages in 30-second epochs. Currently, the recommended rules are summarized in the Manual for the Scoring of Sleep and Associated Events (Version 3) published by the American Academy of Sleep Medicine (Troester et al., 2023). In older subjects and in patients with sleep disturbances, ambiguous epochs are created by intrusions, translocations, or migrations of specific patterns (Keenan et al., 2013). Consequently, visual sleep scoring, even by well-trained and experienced scorers, retains a degree of subjectivity. Limited interrater reliability is well documented and repeatedly reported (Danker-Hopfe et al., 2004, 2009; Penzel et al., 2013; Rosenberg and Van Hout, 2013; Younes et al., 2016; Cesari et al., 2021; Lee et al., 2022). Recently, we have shown in three different datasets scored by six, nine, and twelve scorers, that sleep stage ambiguity is the rule rather than the exception and that sleep stage probabilities calculated with artificial intelligence (AI) provide an excellent estimate of this ambiguity (Bakker et al., 2023). These sleep stage probabilities, whether based on multiple manual scorings or on autoscoring can be plotted in pseudo colors and have been referred to as hypnodensity graph by Stephansen et al. (2018).

In this topic review we explore how modern AI-based techniques can be used to describe human scoring ambiguity, and how it can be further leveraged to characterize SDB and to unlock the full potential of HSAT. In the first section, we introduce the concept of hypnodensity as a technique to represent the probabilities with which sleep experts assign the 5 sleep stages to each epoch. In the second section, we show how the AI-determined hypnodensity is an excellent estimate of the hypnodensity determined by multiple manual scorings, thus providing an estimate of human ambiguity in sleep scoring. In the third section we provide validation data for hypnodensity-derived sleep staging, and further evaluate the potential benefits of using hypnodensity-derived features to quantify sleep stage ambiguity and stability in patients with sleep apnea. In the fourth section we compare AI-determined sleep stage probabilities estimated from cardiorespiratory signals such as those typically recorded in HSAT, with hypnodensity based on multiple manual scorings. Finally, in the fifth and last section, we show how AI-based scoring of cardiorespiratory signals impacts the agreement between SDB-related sleep parameters derived from reduced montage with those derived from full PSG in patients with sleep disturbances.

## Hypnodensity based on multiple manual scorings

**Figure 1:**
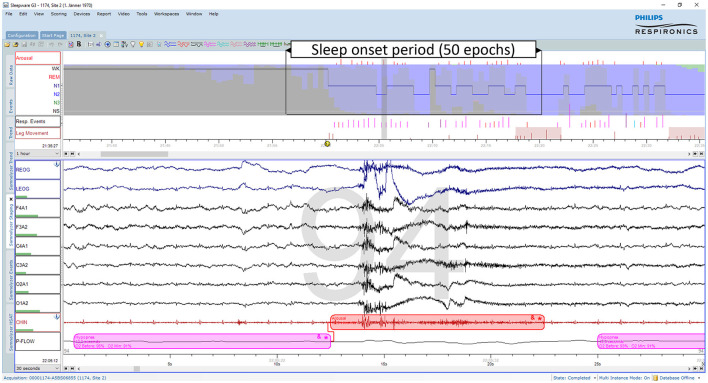
Example of an ambiguous sleep epoch during sleep onset (PSG1: OSAS patient, male, 76 years). Upper part (first hour): The trends from top to bottom are: “Arousals”, arousal events; the hypnogram superimposed on the hypnodensity graph with the color codes W, gray; R, red; N1, cyan; N2, blue; N3, green; “Resp. Events”, respiratory events; “Leg Movements”, leg movement events. Lower part (30-s window): The signals from top to bottom are: “REOG” and “LEOG”, right and left EOG; “F4A1”, “F3A2”, “C4A1”, “C3A2”, “O2A1”, and “O1A2”, the six EEG channels; “CHIN”: chin EMG; “P-Flow”: nasal pressure airflow. This epoch containing an arousal due to a hypopnea was scored as N2 by 7, as N1 by 4 and as W by one out of 12 scorers. Thus, based on 12 manual scorings the sleep stage probabilities for this epoch are for W: 0.08, for N1: 0.33, for N2: 0.58, for N3: 0.0, and for R: 0.0.

**Figure 2:**
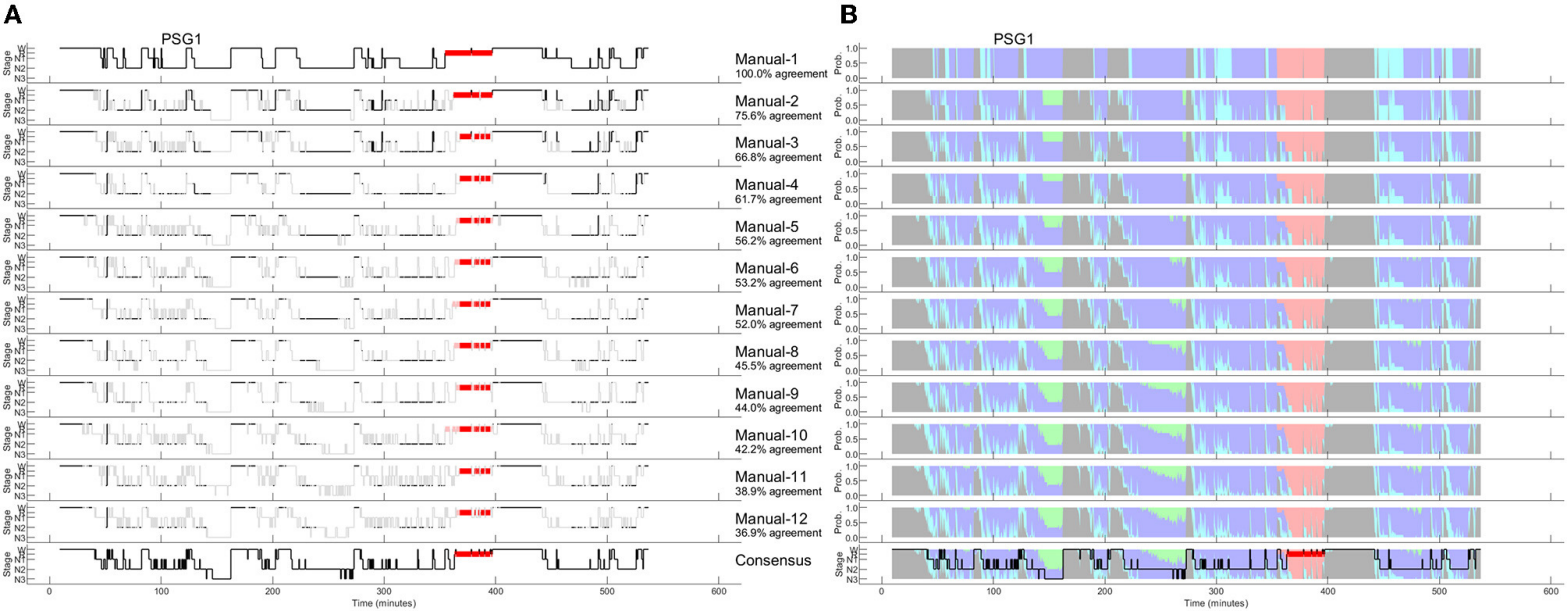
A representative example of 12 manually scored hypnograms and the derived hypnodensities (PSG1: OSAS patient, male, 76 years). **(A)** The individual hypnograms sorted from scorer 1 to scorer 12 (Manual-1 to Manual-12) where epochs with disagreement to the upper scorer(s) are grayed out. The bottom hypnogram depicts the consensus scoring based on majority vote. **(B)** The corresponding hypnodensity graphs based on the sorted scorings (Manual-1 to Manual-12). Thus, the first hypnodensity graph is based on Manual-1 scoring only, the second on Manual-1 and Manual-2 scorings, etc. The (two) last hypnodensity graphs are based on all 12 manual scorings where the consensus hypnogram is superimposed on the last hypnodensity. The color codes are W, gray; R, red; N1, cyan; N2, blue; N3, green. Note that while the agreement between the first two scorers is 75.6%, the agreement decreases continuously with each new scorer included in the comparison so that if 12 scorers are compared the percentage of epochs with complete agreement is reduced to 36.9%.

As discussed recently by Penzel (2022), error rates of 15% or more are usually accepted for sleep stage scoring. The author stated that an agreement between sleep stage scorers of 85% is acceptable, and it gets worrying if the agreement drops below 70%. However, these values only apply to the comparison between two scorers. As three or more scorers are compared, the percentage of complete agreement between the scorers continues to decrease (Bakker et al., 2023). Figure 1 shows an example of a 30-second epoch with ambiguity, which can be uncovered by independently assessing the study by multiple scorers. The example shown in Figure 1 was taken from a study with independent scorings by 12 human experts. Seven experts scored this epoch as N2, four as N1 and one scorer scored the epoch as W. In the 50 epochs during the sleep onset period, as indicated by the arrow in the top panel of Figure 1, only 2 epochs were unequivocally scored as W and 4 epochs unequivocally as N2. Thus, during this sleep onset period, the 12 scorers agreed completely on only 12% of the epochs (6 out of 50 epochs). The left part of Figure 2 shows the 12 hypnograms for the study used in the example of Figure 1. The hypnograms are sorted from scorer 1 to scorer 12, and epochs where each scorer disagrees with at least one of the upper scorer(s) are grayed out. As it can be seen, that while scorers 1 and 2 agree for 75.6% of the epochs, the percentage of epochs with complete agreement decreases continuously with each additional scorer. The final set of twelve scorers only reach complete agreement for 36.9% of the epochs (390 out of 1,057 epochs). The right part of Figure 2 presents the sleep stage probabilities as hypnodensity graphs based on aggregated scorers: the first graph corresponds to the first scorer (top graph with probabilities of 0 or 1), the second graph, to the first and second scorers (with probabilities 0 or 1 for epochs with agreement or 0.5 for epochs with disagreement) and so on, until the second graph from the bottom, with the probabilities based on all 12 manual scorings. Note that in this example, not a single epoch of N1 and N3 was scored unequivocally by all 12 experts. Furthermore, only isolated epochs N2 have been scored with complete agreement. Longer periods of complete agreement are mostly found in epochs scored as wake or rapid-eye movement (REM) sleep.

If multiple manual expert scorings are available for a study, it is possible to determine a consensus scoring. In this case, consensus is based on a majority vote. In the 30-second epoch example of Figure 1, the consensus score would be N2 since 7 out of 12 scorers assigned this epoch as N2. To avoid ties, one could weigh the assessments of scorers with a higher agreement with the other scorers (as measured by Cohen's kappa) more than the assessments of scorers with lower agreement (Stephansen et al., 2018). This approach was used in the examples of Figures 2, **4**, resulting in the consensus scorings shown in the bottom left as hypnograms and in the bottom right superimposed on the hypnodensity graphs.

**Figure 3:**
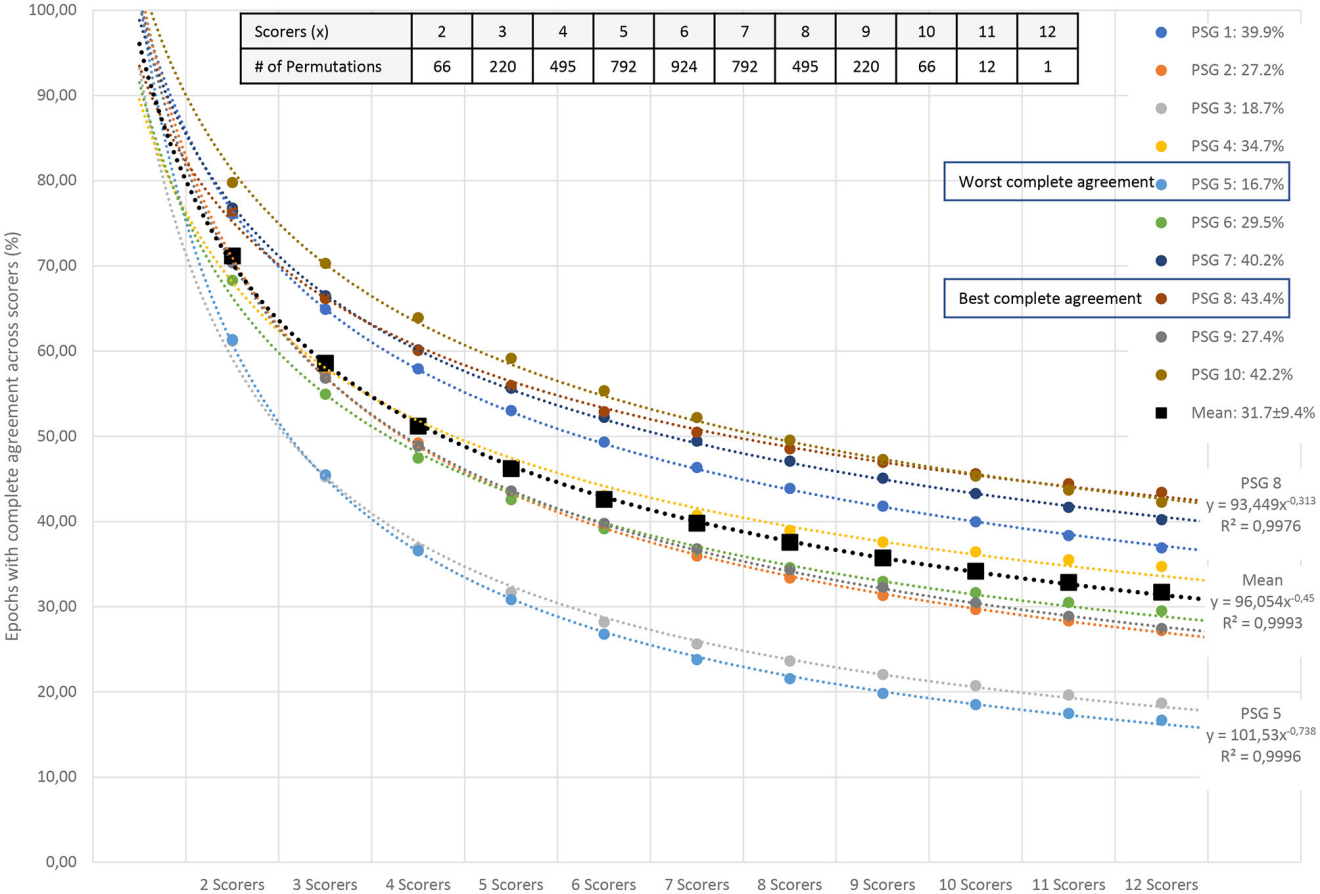
Percentage of all epochs with complete sleep staging agreement across 12 scorers in 10 PSGs. The number of scorers compared is shown on the x-axis; the percentage of complete agreement across the compared scorers is shown on the y-axis. The mean of all possible permutations for choosing x scorers out of the available scorers are shown for each PSG (colored filled circles) and for the mean of the 10 PSGs (black filled squares). The table in the upper right corner describes the number of permutations for each combination of scorers. The reduction in complete agreement alongside the increasing number of scorers follows an almost-perfect power function (dashed lines for each dataset). In addition to the coefficients and exponents, the explained variances (R^2^) are shown. Modeled together, the power function y = ax^b^ has a coefficient a_MEAN_ of 96 and an exponent b_MEAN_ of−0.45. This function explains more than 99% of the variance.

**Figure 4:**
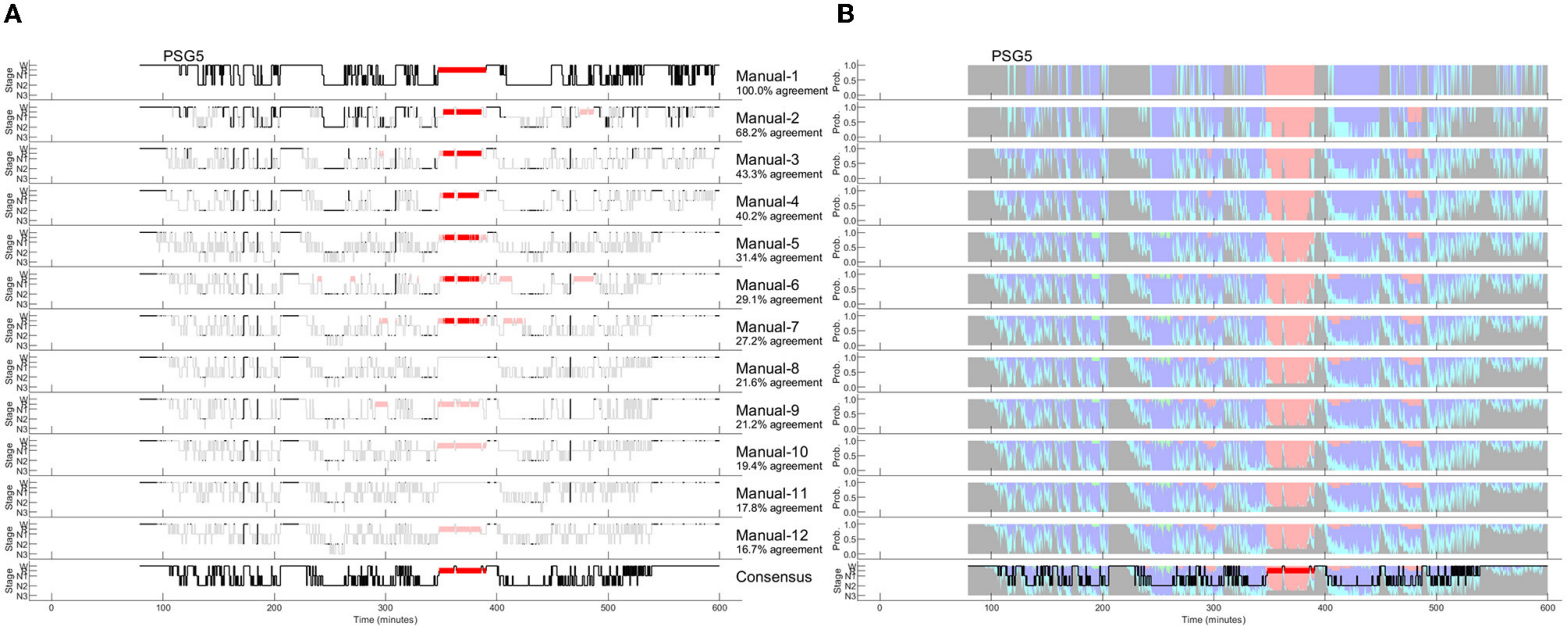
Manually scored hypnograms and the derived hypnodensities for the study with the worst agreement between the 12 scorers (PSG5: Hypersomnia with sleep apnea, male, 78 years). **(A)** The individual hypnograms sorted from scorer 1 to scorer 12 (Manual-1 to Manual-12) where epochs with disagreement to the upper scorer(s) are grayed out. The bottom hypnogram depicts the consensus scoring based on majority vote. **(B)** The corresponding hypnodensity graphs based on the sorted scorings (Manual-1 to Manual-12). Thus, the first hypnodensity graph is based on Manual-1 scoring only, the second on Manual-1 and Manual-2 scorings, etc. The (two) last hypnodensity graphs are based on all 12 manual scorings where the consensus hypnogram is superimposed on the last hypnodensity. The color codes are W, gray; R, red; N1, cyan; N2, blue; N3, green. Note that while the agreement between the first two scorers is 68.2%, the agreement decreases continuously with each new scorer included in the comparison so that if 12 scorers are compared the percentage of epochs with complete agreement is reduced to 16.7%.

Since the amount of agreement progressively displayed in Figure 2 depends on the order of the scorers, we performed all possible order permutations across the twelve scorers and averaged the percentages of epochs with complete agreement. Figure 3 shows the averaged percentages of complete agreement across scorers for all 10 studies with 12 scorings, vs. the number of scorers compared. The number of permutations which depends on the number of scorers compared is shown in the table on the top. Interestingly, the decline in agreement can be modeled almost perfectly by a power function y = ax^b^ where y is the percentage of epochs with complete agreement, x is the number of scorers, a is the coefficient (in %), and b is the exponent. This model explained almost 100% of the variance not only for PSG 1 (blue line, corresponds to the study illustrated in Figure 2) but for each of the 10 PSGs (all R^2^ > 0.99) with a constant close to 100% and an exponent between −0.31 and −0.74, depending on the study. The worst complete agreement between the 12 scorers (16.7%) was found for PSG 5, where like for PSG 3, the agreement already falls below 50% when comparing three scorers. Figure 4 illustrates the study PSG 5 in the same way as PSG 1 was illustrated in Figure 2. Note that there are only a few isolated epochs of N2 and some epochs of W that have been scored unequivocally by the 12 experts. In Bakker et al. (2023), we described that the power functions were very similar also for two other datasets with 70 PSGs scored by 6 scorers and 15 PSGs scored by nine scorers, indicating a robust effect independent of the dataset and the scorers. On average, the exponent of the power function is close to −0.5 indicating that the scoring agreement is approximately inversely proportional to the square root of the number of scorers included in the analysis. This means that on average, the complete agreement drops to 50% when four or more scorers are compared [100%/sqrt(4) = 50%].

To derive the AASM-recommended sleep parameters, one can use the consensus hypnogram. Alternatively, all the parameters could be determined from each of the 12 hypnograms and subsequently averaged to achieve a more robust estimate of the patients' sleep characteristics. Table 1 compares the results for both approaches for the example study shown in Figure 2 and demonstrates once more the impact of between-scorer variability on the derived sleep parameters. As shown in Table 1, the total sleep time varies between 348 and 408 min, the time spent in N1 varies between 23.5 and 134 min, the time in N2 varies between 180 and 311.5 min, the time in N3 varies between 0 and 67 min and the time in R varies between 22 and 42 min, depending on which expert scored this study. Averaged over the 10 studies with 12 scorers, total sleep time varied between 319 and 390 min, the time in N1 varies between 29 and 127 min, the time in N2 varies between 125 and 250, the time in N3 varies between 7 and 56 min, and the time in R varies between 42 and 63 min. Since each expert typically interprets the rules consistently, paired samples t-tests comparing the parameters based on the two extreme values are all significant at *p* < 0.01. Moreover, significant differences in the total sleep time, and the time spent in each of N1, N2, N3, and R were also found for many of the 66 possible pairwise comparisons between two scorers. For example, 15 scorer pairs differed significantly at *p* < 0.01 in the scoring of total sleep time. The number of significant *t*-tests were 25, 28, 27, and 9 for the time spent in N1, N2, N3, and R, respectively. Only 5 out of the 66 scorer pairs showed no significant difference in any of the 5 parameters (i.e., scorer 1 vs. scorer 2, scorer 1 vs. scorer 6, scorer 2 vs. scorer 3, and scorer 6 vs. scorer 7).

**Table 1:** Sleep parameters derived from multiple manual scorings (OSAS patient, male, 76 years).

**Scorer**	**TST (min)**	**N1 (min)**	**N2 (min)**	**N3 (min)**	**R (min)**
Manual-1	356.5	83	231.5	0	42
Manual-2	381.5	74.5	253	21	33
Manual-3	357.5	88	247	0	22.5
Manual-4	387	48	311.5	0	27.5
Manual-5	408	94.5	264	18.5	31
Manual-6	360	53.5	245.5	33.5	27.5
Manual-7	382	83.5	248	19.5	31
Manual-8	370.5	45	228.5	67	30
Manual-9	370.5	23.5	255.5	62.5	29
Manual-10	399	115.5	200	45	38.5
Manual-11	348	96	217	13	22
Manual-12	375.5	134	180	39	22.5
Manual-Mean = Manual-Hypnodensity	374.7	78.3	240.1	26.6	29.7
Manual-Consensus Hypnogram	372.5	62	259	20.5	31

Manual-1 to Manual-12: parameters derived from the twelve individual manual scorings. Manual-Mean: mean value of the 12 parameters, which is equal to parameters derived from the hypnodensity based on the manual scorings. Manuel-Consensus Hypnogram: parameters derived from the manual consensus hypnogram. Light and dark ocher cells indicate per parameter the lowest and highest value, respectively; gray cells indicate parameters based on multiple manual scorers.

Similar high inter-scorer ranges have been reported by Magalang et al. (2013) averaged over 15 PSGs scored by 9 experts (N1: 32 to 111 min; N3: 25 to 73 min) as well as by Younes et al. (2018) averaged over 70 PSGs scored by 10 experts (N1: 16 to 155 min; N3: 4 to 111 min). The consequences of these different interpretations are certainly significant. Younes et al. (2018) stated that in nearly all the 70 PSGs, regardless of the average value obtained from the 10 scorers, stage N1 sleep time could be reported as well below normal or well above normal just depending on who scored the PSG. Similarly, reported stage N3 sleep time ranged from zero to high values regardless of average stage N3 sleep time of the PSG.

To avoid individual scorer bias in the estimation of sleep parameters, one may make use of multiple expert scorings. The averaged parameters are given in the penultimate row of Table 1 (Manual-Mean). Alternatively, these averaged parameters can be computed directly from the hypnodensity graph by calculating the area under the sleep stage probability curves of the respective stages (Manual-Hypnodensity). Note that these mean parameters are not necessarily equal to the parameters computed from a majority vote hypnogram. Based on the consensus hypnogram (last line in Table 1) the time in N1 is 62 min, while the time in N1 is 78.3 min (+16.3 min) if averaged across the times derived from each of the 12 hypnograms or if the N1 probabilities of the hypnodensity curve are integrated. Specifically, the time in N1 is typically underestimated if the parameters are derived from a consensus scoring, since the epoch-by-epoch agreement for stage N1 is generally low between manual scorers. Given the wide variability in the interpretation of the recorded neurological signals by manual experts, it is highly questionable to rely on a single expert's assessment. Furthermore, none of the 12 hypnograms or the consensus hypnogram provide insights into this inter-scorer variability. In contrast, the hypnodensity reflects sleep stage ambiguity while retaining all the information contained in a consensus hypnogram.

## Hypnodensity based on autoscoring using neurological signals

Autoscoring with artificial intelligence enables the direct quantification of sleep stage ambiguity by determining sleep stage probabilities for each epoch. Based on these probabilities, it is possible to create a hypnodensity chart from autoscoring which can be directly compared to the hypnodensity chart based on multiple expert scorings. In Table 2 we summarize publications using AI-algorithms for sleep staging and reporting autoscored hypnodensity graphs (Stephansen et al., 2018; Cesari et al., 2021, 2022; Vallat and Walker, 2021; Anderer et al., 2022b; Brandmayr et al., 2022; Bakker et al., 2023; Fiorillo et al., 2023b). In addition to information regarding the training datasets, the epoch encoder including feature extraction, the sequence encoder and classifier, and the test datasets, the Cohen's kappa values obtained in each study are given. Two publications describe quantitative comparisons between the hypnodensity graph derived from autoscoring vs. the hypnodensity graph derived from multiple manual scorings. Bakker et al. (2023) computed the intra-class correlation coefficient (ICC) for absolute agreement between the probability curves from auto and manual scoring per sleep stage as well as overall sleep stages for the entire night, while Fiorillo et al. (2023b) computed the cosine similarity between the probability values from auto and manual scoring per 30-s epoch and averaged these values over the entire night resulting in average cosine similarity (ACS) measures. As can be seen in Table 2, both approaches indicate a high agreement between the autoscored and the manually derived hypnodensity graphs with an ICC of 0.91 and an ACS of up to 0.91.

**Table 2:** Overview of sleep staging validation studies with algorithms outputting the hypnodensity graph.

**Author, year, algorithm name**	**Training dataset**	**Feature extraction /** **epoch encoder**	**Classifier/Sequence encoder**	**Test dataset**	**Cohen's κ**	**Hypnodensity**
Stephansen et al. (2018) *Stanford-STAGES*	Cohort: *N =* 2,784; 10 cohorts	EEG, EOG, EMG: Cross-correlation encoding+CNN	Unidirectional LSTM	Cohort: *N =* 70; IS-RC, 6 scorers	0.75	
Cesari et al. (2021) *Stanford-STAGES*	Stanford-STAGES algorithm			Cohort: *N =* 1,066; SHIP, 2 scorers	0.68	
Vallat and Walker (2021) *YASA*	Cohort: *N =* 3,163; 7 cohorts	EEG, EOG, EMG (time-domain features and spectrogram)	LightGBM	HC: *N =* 25; DOD-H, 5 scorersPAT(OSA): *N =* 55; DOD-O, 5 scorers	0.80 0.77	
Anderer et al. (2022b) *Somnolyzer*	Cohort: *N =* 588; SIESTA (7 sleep centers) 2–6 scorers	EEG, EOG, EMG (Sleep/wake related features) + MLP/CNN	bidirectional LSTM ( → RandK) + CNN + unidirectional LSTM ( → AASM)	Cohort: *N =* 426; ABC, homePAP, MESA, 1 scorer	0.74	
Bakker et al. (2023) *Somnolyzer*	Somnolyzer algorithm			Cohort: *N =* 70; IS-RC, 6 scorersPAT: *N =* 15; SAGIC, 9 scorersPAT: *N =* 10;Somnoval,12 scorers	0.780.75 0.76	ICC: 0.91ICC: 0.91 ICC: 0.91
Cesari et al. (2022) *Stanford-STAGES*	Stanford-STAGES algorithm			PAT (Narcolepsy type1 and 2, idiopathic hypersomnia, subjective EDS): *N =* 143, 1 scorer	0.75	
Cesari et al. (2022) *YASA*	YASA algorithm				0.76	
Brandmayr et al. (2022) *ENGELBERT*	HC: *N =* 20;Sleep-EDF-20 HC: *N =* 78;Sleep-EDF-SC Cohort: *N =* 62;MASS-SS3	Single-channel EEG (raw signal) + CNN	Local MHSA on overlapping windows	HC: *N =* 20; Sleep-EDF-20 (CV) HC: *N =* 78; Sleep-EDF-SC (CV) Cohort: *N =* 62; MASS-SS3 (CV)	0.82 0.79 0.80	
Fiorillo et al. (2023b) *DeepSleepNet-Light*	Cohort: *N =* 70; IS-RC, 6 scorersHC: *N =* 25; DOD-H, 5 scorers PAT (OSA): *N =* 55; DOD-O, 5 scorers	Single-channel EEG (raw signal) + CNN	Deep CNN (Soft consensus label smoothing)	Cohort: IS-RC, 6 scorers (CV)HC: DOD-H, 5 scorers (CV) PAT (OSA): DOD-O,5 scorers (CV)	0.67 0.76 0.71	ACS: 0.85ACS: 0.91 ACS: 0.89
Fiorillo et al. (2023b) *Simple Sleep Net*		EEG, EOG, EMG (spectrogram) + bidirectional GRU + Attention Layer	Bidirectional GRU (Soft consensus label smoothing)	Cohort: IS-RC, 6 scorers (CV) HC: DOD-H, 5 scorers (CV) PAT (OSA): DOD-O,5 scorers (CV)	0.73 0.84 0.80	ACS: 0.82ACS: 0.91 ACS: 0.91

Cohort, cohort study; HC, healthy controls; PAT, patients with sleep disturbance; OSA, patients with obstructive sleep apnea; PD, Parkinson's Disease; IS-RC, Research study on sleep disordered breathing in women in midlife, University of Pennsylvania, Philadelphia; SIESTA, SIESTA sleep database; Somnoval, Somnolyzer validation study dataset; Sleep-EDF, Sleep-EDF Database Expanded; DOD-H, Dreem Open Dataset – Healthy Volunteers; DOD-O, Dreem Open Dataset – Obstructive Sleep Apnea Patients; homePAP, Home Positive Airway Pressure study; ABC, Apnea, Bariatric surgery, and CPAP study; SHIP, Study of Health in Pomerania; MESA, Multi-Ethnic Study of Atherosclerosis; SAGIC, Sleep Apnea Genetics International Consortium, The Ohio State University Medical Center, Columbus; CNN, Convolutional neural network; LSTM, long short-term memory; MLP, multilayer perceptron; LightGBM, decision tree-based gradient-boosting machine; decision tree; MHSA, Multi-head self-attention.

The Stanford-STAGES, YASA, Somnolyzer and Simple Sleep Net algorithms use EEG, EOG and chin EMG channels as inputs, while the ENGELBERT and DeepSleepNet-Light algorithms are based on a single EEG channel only, offering sleep staging for reduced montage recordings. The Somnolyzer autoscoring system uses all recorded frontal, central and occipital EEG channels, left and right EOG channels, as well as the chin EMG channel for feature extraction. Somnolyzer feature extraction includes identification of artifacts, detection of slow waves, k-complexes, sleep spindles, and episodes with alpha waves, determination of the EEG background activities (delta, theta, alpha, slow and fast beta activities) based on the EEG channels. EOG channels were used for detecting slow and rapid eye movements as well as eye-blinks. The chin EMG channel was used to detect tonic and transient EMG activities (Anderer et al., 2005, 2010). The original Somnolyzer algorithm (Version 1.7; 2005) was developed according to the criteria defined by Rechtschaffen and Kales (1968) (R&K) and subsequently modified (Version 1.8; 2009) to comply with the AASM 2007 criteria (Iber et al., 2007). For version 4.0 of Somnolyzer released commercially in 2021, a supervised deep learning algorithm was used to train a neural network where 472 PSGs from the SIESTA database (Klosch et al., 2001) were used for parameter optimization, and the remaining 116 PSGs were used for early-stopping to prevent the model from overfitting. Each PSG was scored by two independent technologists and one consensus scorer chosen from a pool of 30 scorers to obtain R&K sleep stage probabilities as training targets. This corresponds to the soft consensus labels smoothing approach using an alpha coefficient of 1 as presented by Fiorillo et al. (2023b). The categorical cross-entropy between the sleep stage probabilities and the network output with softmax activation was used as loss function during the training. In a further step, arousals, sleep spindles, and k-complexes were added to the feature set and a convolutional neural network (CNN) followed by a bidirectional long short-term memory (LSTM) layer was trained using data from 72 PSGs scored according to AASM criteria to sub-classify NREM sleep stages.

**Figure 5:**
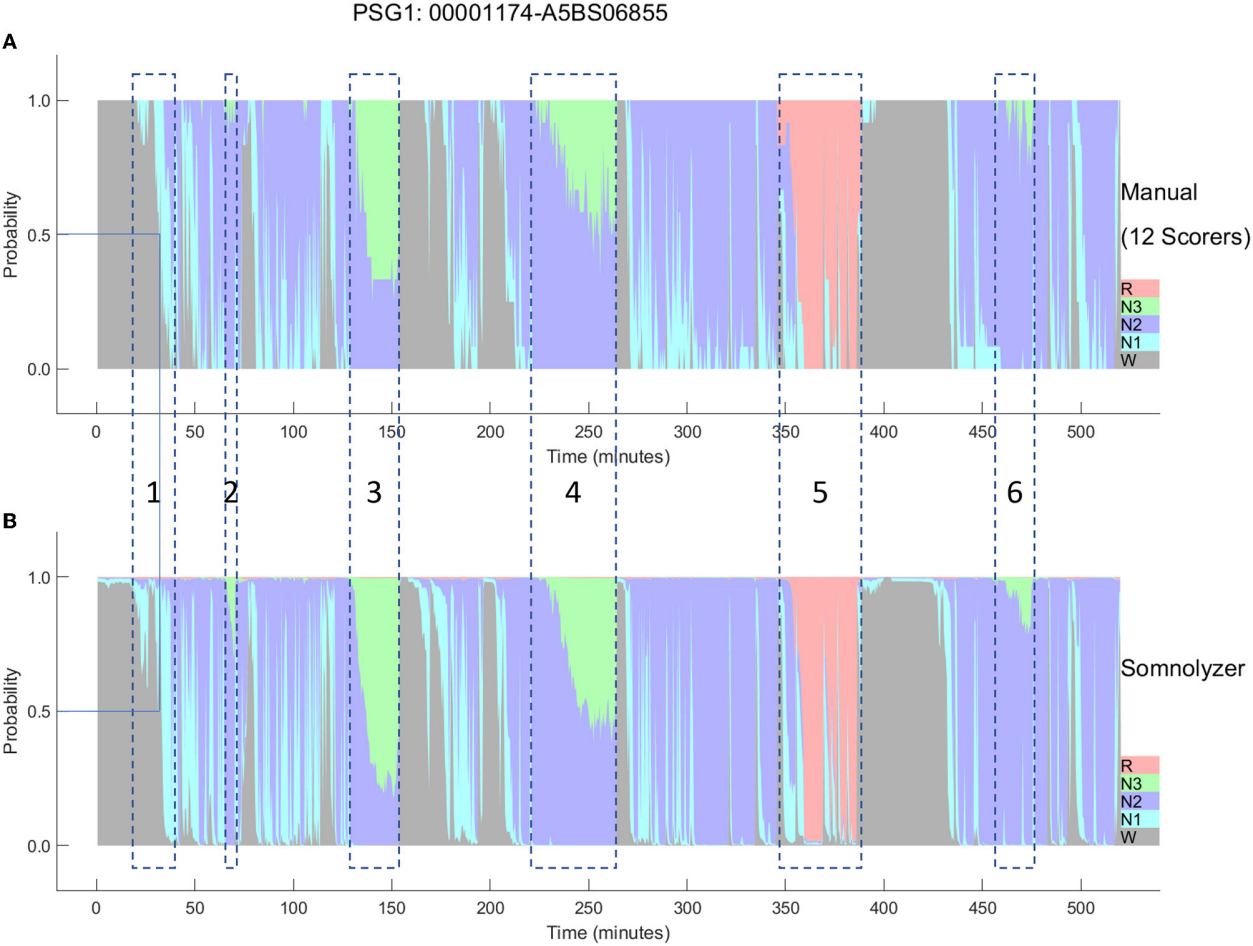
A representative example comparing the hypnodensities derived from 12 manual scorings **(A)** and from autoscoring **(B)** for the same PSG shown in Figure 2 (PSG1: OSAS patient, male, 76 years). The color codes are W, gray; R, red; N1, cyan; N2, blue; N3, green. The time period depicted in box 1 highlights the sleep onset period with the actual sleep onset at W_prob_ <0.5 indicated as solid line; Boxes 2, 3, 4, and 6 indicate time periods where at least 2 scorers have scored N3; Box 5 indicate the time period where at least one scorer has scored R. Note the similarity of the manually-derived and the autoscoring-derived sleep stage probabilities.

**Figure 6:**
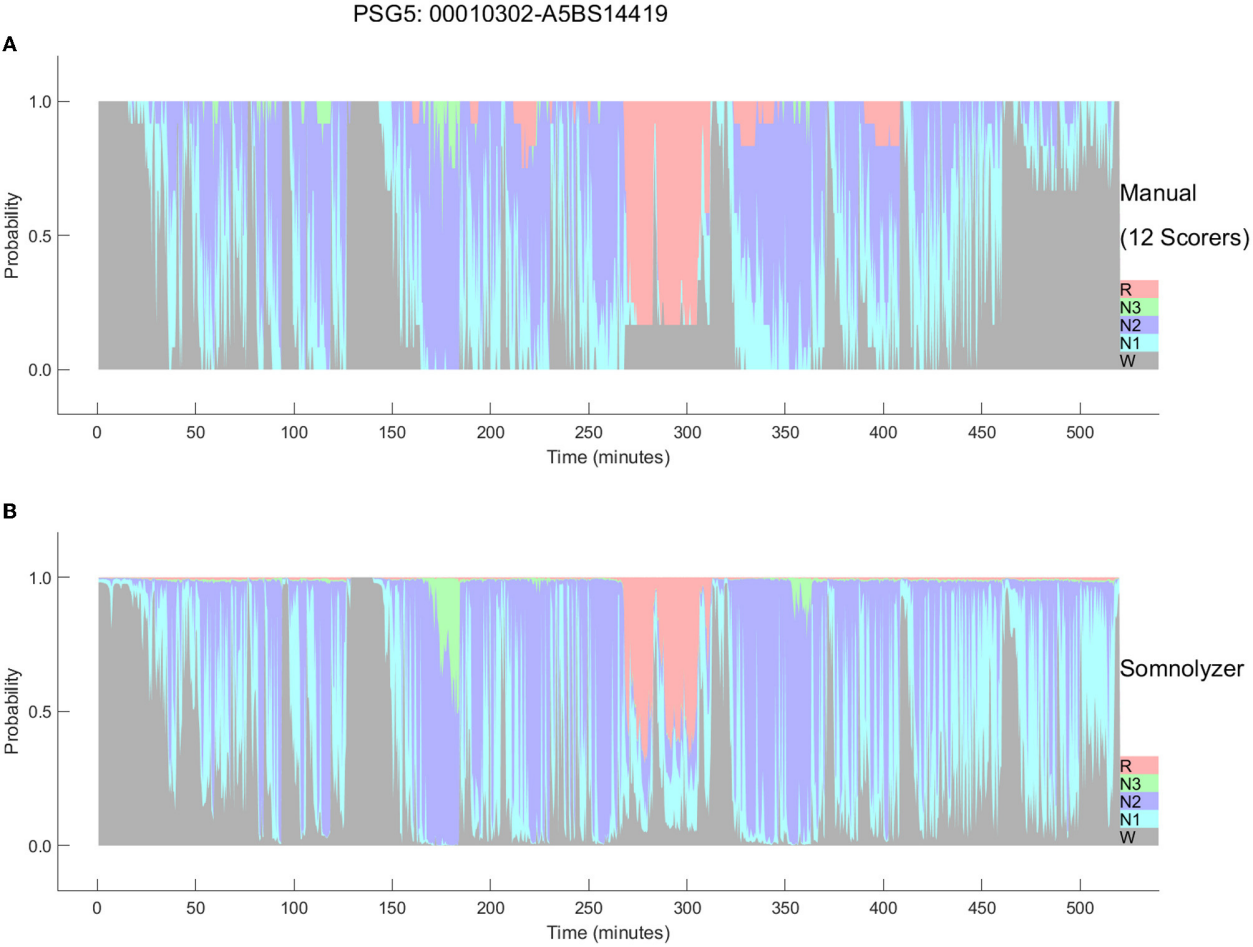
Comparison between the hypnodensities derived from 12 manual scorings **(A)** and from autoscoring **(B)** for the PSG with the worst agreement between manual scorers (PSG5: Hypersomnia with sleep apnea, male, 78 years). The color codes are W, gray; R, red; N1, cyan; N2, blue; N3, green. Note the similarity of the manually-derived and the autoscoring-derived sleep stage probabilities.

The final Somnolyzer network output assigned AASM-related sleep stage probabilities of W, N1, N2, N3, R to each 30-sec epoch. Figures 5, 6 compare the hypnodensity derived from the 12 manual scorings with the hypnodensity determined by the Somnolyzer autoscoring system for the two studies shown in Figures 2, 4, respectively. As can be seen in the examples from this independent test set, the sleep stage probabilities of Somnolyzer match almost perfectly with the sleep stage probabilities based on the 12 human scorings. The ICCs for absolute agreement between the two probability curves are 0.97 for PSG 1 (Figure 5) and 0.89 for PSG 5 (Figure 6). Also note the high similarity in the probability curves for each sleep stage. In Figure 5, we highlighted 6 periods. Box 1 comprises sleep onset with a change from W to a small amount of N1 probability at the start, back to definite W, and then with increasing N1 probability via sleep onset, which is the first epoch with sleep probability higher than wake probability (solid line), to definite sleep with N1+N2 probability above 0.95 with approximately equal amount of N1 and N2 probability at the end of the box 1. Note that just before the end of box 1, there are epochs which were assigned still as W by some scorers, while others assigned these epochs already as N2 sleep. Boxes 2, 3, 4, and 6 indicate periods where at least two experts scored N3. Note that the N3 probabilities derived from autoscoring follow the N3 probabilities derived from the 12 manual scorings not only in respect to the timing, but also in respect to the amount, with the highest N3 probability reaching 0.75 (8 out of 12 scorers) in box 3 and the lowest at 0.17 (2 out of 12 scorers) in box 2. Finally, also for the R probabilities (box 5) the manually- and autoscoring-based curves match in terms of time and magnitude. Interestingly, even for the study with the worst agreement between scorers, the manually- and autoscoring-based probability curves match closely (Figure 6).

As presented in Bakker et al. (2023) the ICCs for absolute agreement between sleep stage probabilities derived from manual- and Somnolyzer autoscoring were, on average 0.91 for all stages for all three datasets with multiple scorers (Table 2). For the individual sleep stages the ICCs were as follows: 0.93-0.94 for stage W; 0.72–0.74 for stage N1; 0.88–0.89 for stage N2; 0.85–0.94 for stage N3; and 0.96–0.97 for stage R. Thus, according to the thresholds defined by Koo and Guideline (2016) the probability curves for all stages, as well as for individual stages W and R show excellent agreement; good agreement for stages N2 and N3; and, moderate agreement even for stage N1.

The hypnodensity graph based on the manual scorers shown in Figure 5 clearly indicates that although all experts follow the well-established AASM rules for scoring PSGs, the interpretation of the rules may, and often does vary substantially between scorers, specifically for epochs or events with equivocal features (Rosenberg and Van Hout, 2013; Younes et al., 2016, 2018). Experts, when scoring these epochs, may be biased toward sensitivity or specificity, probably depending on their internal representation of the features (i.e., their personal feature template or prototype). As soon as the features in the epoch are close enough to their subjective template, the scorer will score this epoch accordingly. Consequently, if the following epoch shows features that are similar or even closer to their template, the scorers will continue to score the same sleep stage. This also explains the gradual increase in epochs scored as sleep (box 1) or in epochs scored as N3 (boxes 3 and 4). If the features never match close enough their personal template of slow wave sleep, scorers may never even start scoring N3 in the entire recording, which is the case for scorers 1, 3, and 4 in the recording shown in Figure 5 (see also Table 1). In contrast, scorers 8 and 9 have obviously a very sensitive template of slow wave sleep resulting in 4 periods of slow wave sleep with a total time in N3 of more than 1 h (Table 1). Younes et al. (2018) showed that some technologists scored stage N3 sleep when delta wave duration was well below 6 s whereas for others much greater durations were required.

Interestingly, by varying sensitivity settings, an autoscoring can mimic these different interpretations. The Somnolyzer autoscoring system has the option to select different sensitivities for arousal, spindle/k-complex, slow wave, apnea and hypopnea event detection. In Anderer et al. (2022b) we reported the effects of changing these sensitivity settings in a study based on 10 PSGs from 10 apnea patients (5 diagnostic-, 2 titration- and 3 split-nights) each scored independently by 8 experts. Sleep parameters derived from the manual scorings varied considerably between the 8 scorers (time in N1: 29–127 min, time in N2: 125–209 min, time in N3: 19–56 min, time in R: 42–63 min). With the default (= balanced) setting, Somnolyzer autoscoring was close to the mean of the 8 manual scorings (time in N1: 82 and 85 min, time in N2: 184 and 176 min, time in N3: 41 and 42 min, time in R: 59 and 56 min, for the Somnolyzer scoring and the mean of the 8 manual scorings, respectively). Moreover, by varying the sensitivity settings in 5 steps (from maximal precision to maximal sensitivity), the autoscoring perfectly mimicked the variability observed in the 8 manual scorers. Thus, by merely varying the sensitivity settings, the inter-scorer variability observed in manual scorings can be explained. Furthermore, the high agreement between the hypnodensity based on the manual scorings and the autoscoring indicates that the 30 scorers who participated in the scoring of our training set covered the full spectrum from maximal sensitivity to maximal precision. In a recent paper on interpretation and further development of the hypnodensity, Huijben et al. (2023) concluded based on theoretical analyses and empirical evidence that the hypnodensity graph, predicted by a classifier that had been trained in a supervised manner, resembles the inter-rater disagreement across the scorers that annotated the PSGs of the training set. Consequently, training sets used to develop classifiers for sleep staging have to be scored by experts covering the full spectrum from highly sensitive to highly precise within the AASM scoring rules in a sufficiently large sample of subjects of both sexes, including healthy controls and a wide range of patients with different sleep disturbances, to ensure that the hypnodensity output of the trained neural network reflects this full spectrum.

## Hypnodensity-derived sleep stages and parameters

Based on the hypnodensity, a sleep stage can be assigned as the stage with the highest probability (argmax). The Somnolyzer algorithm, however, uses a hierarchical approach to assure that for instance epochs with a higher probability for sleep than for wake are scored as sleep, even if the probability for wake is higher than for any individual sleep stage. In the hierarchical approach a sleep stage is assigned to each epoch as follows: if the wake-probability was > 0.5, assign W; else if the REM-probability was higher than the NREM probability (sum of the probabilities of N1, N2, and N3), assign R; else if the N3-probability was higher than the sum of the N1- and N2-probabilities, assign N3; else if the N2-probability was higher than the N1-probability, assign N2; otherwise assign N1. In an additional post-processing step, Somnolyzer enforces the AASM smoothing rules for scoring R as well as N2, based on the occurrence (start and duration) of arousals, sleep spindles and K complexes (Troester et al., 2023).

A validation study of the Somnolyzer algorithm by Anderer et al. (2022b) with 426 PSGs (224 PSGs from the MESA study (Chen et al., 2015), 178 PSGs from the HomePAP study (Rosen et al., 2012) and 24 PSGs from the ABC study (Bakker et al., 2018) scored by one scorer resulted in a Cohen's kappa between Somnolyzer autoscoring and manual scoring of 0.739 (with a 95% confidence interval of 0.737 to 0.741), reflecting substantial agreement according to the thresholds defined by Landis and Koch (1977). In agreement with human inter-rater reliability studies, the highest kappa values were observed for wake and REM detection (0.85 and 0.87) followed by N2 and N3 detection (0.72 and 0.73), while the detection of N1 resulted in the lowest kappa value of 0.46.

Another Somnolyzer validation study based on the three external datasets scored by six, nine, and twelve scorers, demonstrated for each dataset that the agreement between autoscoring and consensus manual-scoring was significantly higher than agreement between manual-scoring and consensus manual-scoring (Bakker et al., 2023). In the dataset with 70 PSGs and 6 scorers, autoscoring achieved a Cohen's kappa of 0.78, vs. 0.69 for manual scorings; for the dataset with 15 PSGs and 9 scorers, autoscoring achieved 0.74 vs. 0.67 for manual scorings; and for the dataset with 10 PSGs and 12 scorers, 0.75 vs. 0.67 (all *p* < 0.01). As shown by the authors in supplementary tables, the percentage of agreement between autoscoring and consensus scoring was 85, 83 and 83% for the three studies. Thus, in 15–17% of the epochs, Somnolyzer disagreed with the consensus. However, in almost all of these epochs at least one scorer disagreed with the consensus and more importantly, autoscoring agreed in these cases with at least one of the deviating scorers. By considering as a correct detection, all epochs where autoscoring and at least one manual scorer agreed, the percentage of agreement increases to 97.9, 98.3, and 99.1% for the three datasets with 6, 9, and 12 scorers. In addition, the authors showed that sleep staging derived from autoscoring was for each individual PSG non-inferior to manual-scoring.

Many AI-based sleep scoring algorithms have been developed and validated in the last few years. In Tables 3, 4 we summarize publications using AI-algorithms for sleep staging based on neurological signals that reported Cohen's kappa for the 5-stage comparison. Note the large difference in the size of the training data (between 10 and more than 15,000 PSGs) as well as in the size of the test data (between 8 and close to 3000). Table 3 summarizes validation results of AI-algorithms that applied a hold-out or cross-validation; i.e., an internal validation based on data from the same dataset that has been used for training (Supratak et al., 2017; Sors et al., 2018; Phan et al., 2019; Zhang et al., 2019; Abou Jaoude et al., 2020; Guillot et al., 2020; Korkalainen et al., 2020; Sun et al., 2020a; Alvarez-Estevez and Rijsman, 2021; Fiorillo et al., 2021, 2023b; Jia et al., 2021; Nasiri and Clifford, 2021; Olesen et al., 2021; Pathak et al., 2021; Vallat and Walker, 2021; Brandmayr et al., 2022; Cho et al., 2022; Ji et al., 2022; Li C. et al., 2022; Li T. et al., 2022; Sharma et al., 2022; Yubo et al., 2022). Table 4 summarizes the validation results of AI-algorithms which have been validated in datasets completely unseen by the model (Anderer et al., 2018, 2022b; Biswal et al., 2018; Patanaik et al., 2018; Stephansen et al., 2018; Zhang et al., 2019; Abou Jaoude et al., 2020; Alvarez-Estevez and Rijsman, 2021; Cesari et al., 2021, 2022; Vallat and Walker, 2021; Bakker et al., 2023). The reported Cohen's kappa values ranged between 0.60 [external validation in 70 patients with Parkinson's disease (Patanaik et al., 2018)] to 0.91 [internal 20-fold epoch-wise cross validation in 8 healthy subjects (Li C. et al., 2022)]. Cohen's kappa values reported for the 45 internal validation studies using hold-out or cross-validation were significantly higher than for the 19 studies using external test sets for validation (0.79 ± 0.04 vs. 0.72 ± 0.06; *p* < 0.001 independent samples *t*-test). In all studies that reported Cohen's kappa for both, an internal and an external test set the kappa for the internal testing was always higher than for the external testing (Zhang et al., 2019; Abou Jaoude et al., 2020; Alvarez-Estevez and Rijsman, 2021; Vallat and Walker, 2021). Moreover, in studies reporting Cohen's kappa for patients and for healthy controls, the kappa for controls was always higher than for patients (Supratak et al., 2017; Guillot et al., 2020; Korkalainen et al., 2020; Vallat and Walker, 2021; Ji et al., 2022; Yubo et al., 2022). Accordingly, Korkalainen et al. (2020) reported a decrease in Cohen's kappa depending on OSA severity in a clinical dataset of 891 patients, with a kappa of 0.79 for individuals without OSA diagnostic (*n* = 152) to a kappa of 0.68 for patients with severe OSA (*n* = 254). Consequently, performance measures of sleep stage validation studies need to be interpreted depending on the validation method used and the characteristics of the subjects included in the test dataset.

**Table 3:** Overview of sleep staging validation studies with AI-algorithms providing Cohen's kappa based on internal validation.

**Author, Year *Name***	**Training dataset**	**Input signals**	**Test dataset**	**Cohen's κ**
Supratak et al. (2017) *DeepSleepNet*	Cohort: *N =* 62; MASS HC: *N =* 20; Sleep-EDF	Single-channel EEG	Cohort: MASS (CV) HC: Sleep-EDF (CV)	0.800.76
Sors et al. (2018)	Cohort: *N =* 5,793; SHHS	Single-channel EEG	Cohort: *N =* 1738; SHHS (HO)	0.81
Phan et al. (2019) *SeqSleepNet*	Cohort: *N =* 200; MASS	EEG, EOG, EMG	Cohort: MASS (CV)	0.82
Zhang et al. (2019)	Cohort: *N =* 5,213; SHHS	EEG, EOG, EMG	Cohort: *N =* 580; SHHS (HO)	0.82
Sun et al. (2020a)	Cohort: *N =* 147; MASS	EEG, EOG, EMG	Cohort: MASS (CV)	0.80
Guillot et al. (2020) *SimpleSleepNet*	HC: *N =* 25; DOD-H, 5 scorersPAT (OSA): *N =* 55; DOD-O, 5 scorers	EEG, EOG, EMG	HC: DOD-H, 5 scorers (CV) PAT (OSA): DOD-O, 5 scorers (CV)	0.850.82
Korkalainen et al. (2020)	HC: *N =* 153; Sleep-EDF PAT (OSA): *N =* 891; Clinical Dataset	Single-channel EEG (and single-channel EOG)	HC: Sleep-EDF (CV) PAT (OSA): Clinical datsaset (CV)	0.78 0.78
Abou Jaoude et al. (2020)	PAT: *N =* 6,341; MGH	4 EEG channels	PAT: *N =* 791; MGH (HO)	0.74
Alvarez-Estevez and Rijsman (2021)	Cohort: *N =* 443; 6 datasets	EEG, EOG, EMG (raw signal) + CNN	Cohort: *N =* 88 (HO)	0.80
Olesen et al. (2021)	Cohort: *N =* 15,684; 5 cohorts	EEG, EOG, EMG	Cohort: *N =* 1,584 (HO)	0.80
Vallat and Walker (2021) *YASA*	Cohort: *N =* 3,163; 7 cohorts	EEG, EOG, EMG	Cohort: *N =* 585 (HO)	0.82
Nasiri and Clifford (2021)	PAT: *N =* 994; PhysioNet	6 EEG channels	PAT: PhysioNet (CV)	0.75
Fiorillo et al. (2021) *DeepSleepNet-Lite*	HC: *N =* 39; Sleep-EDF 2013HC: *N =* 153;Sleep-EDF 2018	Single-channel EEG	HC: Sleep-EDF 2013 (CV) HC: Sleep-EDF 2018 (CV)	0.780.73
Pathak et al. (2021)	Cohort: *N =* 5,793; SHHS PAT: *N =* 1418; Clinical	EEG, EOG, EMG	Cohort: *N =* 579; SHHS (HO) PAT: *N =* 142; Clinical (HO)	0.79 0.68
Jia et al. (2021)	HC: *N =* 10; ISRUC-S3HC: *N =* 62; MASS-SS3	EEG (6 channels ISRUC, 20 channels MASS)	HC: ISRUC (CV) HC: MASS (CV)	0.770.84
Li T. et al. (2022)	HC: *N =* 61; Sleep-EDF 2013 HC: *N =* 197;Sleep-EDF 2018	Single-channel EEG	HC: Sleep-EDF 2013 (CV) HC: Sleep-EDF 2018 (CV)	0.78 0.74
Ji et al. (2022)	HC: *N =* 10; ISRUC-S3 PAT: *N =* 100; ISRUC-S1	EEG, EOG, EMG, ECG	HC: ISRUC-S3 (CV) PAT: ISRUC-S1 (CV)	0.78 0.77
Li C. et al. (2022) *EEGSNet*	HC: *N =* 8; Sleep-EDF-8 HC: *N =* 39; Sleep-EDF-20 HC: *N =* 153; Sleep-EDF-78 Cohort: *N =* 329; SHHS	Single-channel EEG	HC: Sleep-EDF-8 (CV) HC: Sleep-EDF-20 (CV) HC: Sleep-EDF-78 (CV) Cohort: SHHS (CV)	0.91 0.82 0.770.79
Sharma et al. (2022)	Cohort: *N =* 8,455; SHHS	EEG, EOG, EEG	Cohort: *N =* 580; SHHS1 (HO)Cohort: *N =* 2651; SHHS2 (HO)	0.770.80
Cho et al. (2022) *StageNet*	PAT: *N =* 530; Clinical dataset	EEG, EOG, EMG	PAT: *N =* 72, Clinical dataset (HO)	0.84
Yubo et al. (2022) *MMASleepNet*	HC: *N =* 39; Sleep-EDF-20 HC: *N =* 153; Sleep-EDF-78 HC: *N =* 10; ISRUC-Sleep3 PAT: *N =* 100; ISRUC-Sleep1	EEG, EOG, EMG	HC: Sleep-EDF-20 (CV) HC: Sleep-EDF-78 (CV) HC: ISRUC-Sleep3 (CV) PAT: ISRUC-Sleep1 (CV)	0.830.760.77 0.73
Brandmayr et al. (2022) *ENGELBERT*	HC: *N =* 20; Sleep-EDF-20 HC: *N =* 78; Sleep-EDF-SC Cohort: *N =* 62; MASS-SS3	Single-channel EEG	HC: *N =* 20; Sleep-EDF-20 (CV) HC: *N =* 78; Sleep-EDF-SC (CV)Cohort: *N =* 62; MASS-SS3 (CV)	0.820.790.80
Fiorillo et al. (2023b) *DeepSleepNet-Light*	Cohort: *N =* 70; IS-RC, 6 scorersHC: *N =* 25; DOD-H, 5 scorers PAT (OSA): *N =* 55; DOD-O, 5 scorers	Single-channel EEG	Cohort: IS-RC, 6 scorers (CV) HC: DOD-H, 5 scorers (CV) PAT (OSA): DOD-O, 5 scorers (CV)	0.670.76 0.71
Fiorillo et al. (2023b) *Simple Sleep Net*	Cohort: *N =* 70; IS-RC, 6 scorersHC: *N =* 25; DOD-H, 5 scorers PAT (OSA): *N =* 55; DOD-O, 5 scorers	EEG, EOG, EMG	Cohort: IS-RC, 6 scorers (CV) HC: DOD-H, 5 scorers (CV) PAT (OSA): DOD-O, 5 scorers (CV)	0.73 0.840.80
			N-studies	45
			Cohen's κ Mean ± SD	0.79 ± 0.04
			Min	0.67
			Max	0.91

Internal validation, CV, cross-validation; HO, hold-out validation; Cohort, cohort study; HC, healthy controls; PAT, patients with sleep disturbance; OSA, patients with obstructive sleep apnea; MASS, Montreal Archive of Sleep Studies; Sleep-EDF, Sleep-EDF Database Expanded; SHHS, Sleep Heart Health Study; MGH, Massachusetts General Hospital; IS-RC, Research study on sleep disordered breathing in women in midlife, University of Pennsylvania, Philadelphia; DOD-H, Dreem Open Dataset – Healthy Volunteers; DOD-O, Dreem Open Dataset – Obstructive Sleep Apnea Patients; PhysioNet, PhysioNet database resources; ISRUC, ISRUC-SLEEP Dataset.

**Table 4:** Overview of sleep staging validation studies with AI-algorithms providing Cohen's kappa based on external validation.

**Author, Year** ***Name***	**Training Dataset**	**Input Signals**	**Test Dataset**	**Cohen's κ**
Biswal et al. (2018)	PAT: *N =* 9,000; MGH	2-6 EEG channels	Cohort: *N =* 580; SHHS	0.73
Patanaik et al. (2018)	HC: *N =* 1,330; Duke-NUS	2 EEG and 2 EOG channels	PAT: *N =* 210; SDU PAT(PD): *N =* 77; UCSD	0.74 0.60
Stephansen et al. (2018) *Stanford-STAGES*	Cohort: *N =* 2,784; 10 cohorts	EEG, EOG, EMG	Cohort: *N =* 70; IS-RC, 6 scorers	0.75
Anderer et al. (2018) *Somnolyzer*	Cohort: *N =* 588; SIESTA (7 sleep centers) 2–6 scorers	EEG, EOG, EMG	PAT (OSA): *N =* 97; Somnoval, 4 scorers	0.79
Zhang et al. (2019)	Cohort: *N =* 5,213; SHHS	EEG, EOG, EMG	Cohort: *N =* 461; SOF Cohort: *N =* 2,907; MrOS	0.680.70
Abou Jaoude et al. (2020)	PAT: *N =* 6,341; MGH	4 EEG channels	PAT: *N =* 243; homePAP PAT: *N =* 129; ABC	0.69 0.66
Alvarez-Estevez and Rijsman (2021)	Cohort: *N =* 443; 6 datasets	EEG, EOG, EMG	Cohort: *N =* 20-154; inter-cohort performance	0.63
Cesari et al. (2021) *Stanford-STAGES*	Cohort: *N =* 2784; 10 cohorts	EEG, EOG, EMG	Cohort: *N =* 1,066; SHIP, 2 scorers	0.68
Vallat and Walker (2021) *YASA*	Cohort: *N =* 3,163; 7 cohorts	EEG, EOG, EMG	HC: *N =* 25; DOD-H, 5 scorers	0.80 0.77
Anderer et al. (2022b) *Somnolyzer*	Cohort: *N =* 588; SIESTA (7 sleep centers) 2–6 scorers	EEG, EOG, EMG	Cohort: *N =* 426; ABC, homePAP, MESA	0.74
Bakker et al. (2023) *Somnolyzer*	Cohort: *N =* 588; SIESTA (7 sleep centers); 2–6 scorers	EEG, EOG, EMG	Cohort: *N =* 70; IS-RC, 6 scorers PAT: *N =* 15; SAGIC, 9 scorers PAT: *N =* 10; Somnoval, 12 scorers	0.78 0.75 0.76
Cesari et al. (2022) *Stanford-STAGES*	Cohort: *N =* 2,784; 10 cohorts	EEG, EOG, EMG	PAT (hypersomnia): *N =* 143	0.75
Cesari et al. (2022) *YASA*	Cohort: *N =* 3,163; 7 cohorts	EEG, EOG, EMG	PAT (hypersomnia): *N =* 143	0.76
			**N-studies**	**19**
			**Cohen's** **κ** **Mean** **±** **SD**	**0.72** **±** **0.06**
			**Min**	**0.60**
			**Max**	**0.80**

External validation, performance in datasets completely unseen by the model; Cohort, cohort study; HC, healthy controls; PAT, patients with sleep disturbance; OSA, patients with obstructive sleep apnea; PD, Parkinson's Disease; SHHS, Sleep Heart Health Study; MGH, Massachusetts General Hospital; Duke-NUS, Medical School, Singapore; SDU, Sleep Disorders Unit, Singapore General Hospital; UCSD, University of California, San Diego School of Medicine; IS-RC, Research study on sleep disordered breathing in women in midlife, University of Pennsylvania, Philadelphia; SIESTA, SIESTA sleep database; Somnoval, Somnolyzer validation study dataset; SOF, Study of Osteoporotic Fractures; MrOS, Osteoporotic Fractures in Men study; DOD-H, Dreem Open Dataset – Healthy Volunteers; DOD-O, Dreem Open Dataset – Obstructive Sleep Apnea Patients; homePAP, Home Positive Airway Pressure study; ABC, Apnea, Bariatric surgery, and CPAP study; SHIP, Study of Health in Pomerania; MESA, Multi-Ethnic Study of Atherosclerosis; SAGIC, Sleep Apnea Genetics International Consortium, The Ohio State University Medical Center, Columbus.

Table 2 summarizes the kappa values for the 20 validation studies of the 6 AI-based autoscoring algorithms outputting the hypnodensity graph (Stephansen et al., 2018; Cesari et al., 2021, 2022; Vallat and Walker, 2021; Anderer et al., 2022b; Brandmayr et al., 2022; Bakker et al., 2023; Fiorillo et al., 2023b). As can be seen in Table 2, Cohen's kappa for the 5-stage comparison was comparable between the six algorithms. Stephansen et al. (2018), Bakker et al. (2023) and Fiorillo et al. (2023b) validated their algorithms in the same IS-RC cohort and reported, as compared to the consensus of 6 scorers, kappa values between 0.67 and 0.78. Cesari et al. (2022) compared the Stanford-STAGES and YASA algorithm in a dataset of patients with central disorders of hypersomnolence and reported almost identical kappa values for the two algorithms (0.747 and 0.755 for Stanford-STAGES and YASA, respectively). These findings suggest that modern AI-based autoscoring systems offer valid alternatives to manual expert scoring and that the role of manual adjustment and expert review of automatic scorings might no longer be required.

While the most obvious application of the hypnodensity is the determination of the traditional sleep stages, additional information may be derived from the sleep stage probabilities. Stephansen et al. (2018) extracted features from the hypnodensity in patients with narcolepsy by quantifying sleep stage mixing/dissociation. Examples for these features are the time taken before 5% of the sum of the product between W, N2, and REM, calculated for every epoch, has accumulated, weighted by the total amount of this sum or the time taken before 50% of the wakefulness in a rerecording has accumulated, weighted by the total amount of wakefulness. In addition to these hypnodensity-derived features describing unusual sleep stage overlaps, the authors added features expected to predict narcolepsy based on prior knowledge, such as REM sleep latency and sleep stage sequencing parameters. By means of a Gaussian predictor classifier they achieved a specificity of 96% and a sensitivity of 91% for classifying narcolepsy type-1 as validated in independent datasets. In a more recent study, Cesari et al. (2022) investigated whether biomarkers describing sleep instability and architecture derived from both manual hypnogram and automatic hypnogram and hypnodensity graphs might differentiate between distinct disorders of hypersomnolence, such as narcolepsy type-1, narcolepsy type-2, idiopathic hypersomnia and subjective excessive daytime sleepiness. They extracted features from manual and automatic hypnograms, such as standard features, transition features, bouts features, features describing stability and fragmentation of sleep stages as well as the distribution of sleep stages across the night, and REM sleep-specific features such as the number of nightly sleep onset REM periods. In addition, they extracted features from the hypnodensity, including the features proposed by Stephansen et al. (2018) and features reflecting certainty and amount of sleep stages per epoch and across the night. Their results confirmed narcolepsy type-1 specific sleep structure which made it possible to discriminate narcolepsy type-1 from the other groups with high performance (88% accuracy) and narcolepsy type-2 from idiopathic hypersomnia with moderate performance (65% accuracy). Future studies in larger cohorts are needed to improve the differentiation of disorders of hypersomnolence, but these preliminary findings already highlight the promise of hypnodensity in exploiting sleep stage ambiguity and overlap as clinical hallmarks of certain sleep disorders.

In a recent study using the Somnolyzer algorithm (Anderer et al., 2022a), we compared the standard sleep parameters such as total sleep time (TST), sleep latency (SL), REM latency (REML), sleep efficiency (SEFF), wake after sleep onset (WASO), and the time in N1, N2, N3, and R derived from the autoscored hypnogram, to the standard sleep parameters derived from the autoscored hypnodensity in young (20 – <40 years), middle-aged (40 – <60 years) and older (60–95 years) healthy controls from the Siesta database (*n* = 195, 93 males and 102 females aged 20 to 95 years). Overall, the hypnogram-derived and the hypnodensity-derived standard parameters showed very similar age-related changes. However, the age-related changes based on the hypnodensity-derived parameters were consistently slightly higher than the coefficients derived traditionally from the hypnogram.

Moreover, we determined a quantitative measure of sleep stage ambiguity in percent [100^*^(1-p(i)_MAX_) where p(i)_MAX_ is the highest sleep stage probability for epoch i] and a measure of sleep stage continuity in percent [100^*^(1-abs(p(i)_MAX_ - p(i+1)_MAX_)] if epochs i and i+1 are from the same class. In the case of no ambiguity, p(i)_MAX_ is 1 and thus the ambiguity for that epoch is 0%. As can be clearly seen in Figures 5, 6 for both manually-determined and Somnolyzer-determined sleep stage probabilities, the vast majority of epochs have a p(i)_MAX_ <1.0, indicating some amount of ambiguity. In the case of only small changes in the hypnodensity between two adjacent epochs the continuity measure is close to 100%. If the change in the hypnodensity between two adjacent epochs increases, the continuity measure will decrease accordingly. Interestingly, sleep stage ambiguity and continuity showed opposite changes with age for epochs scored as sleep as compared to epochs scored as wake: in wake, ambiguity decreases and continuity increases with age, while during sleep, ambiguity increases, and continuity decreases with age. Together with the significant increase in WASO with age, this means that with increasing age subjects are awake longer, and these wake periods are more definite and stable. On the other hand, during sleep, ambiguity increased, and continuity decreased over age, indicating more uncertainty and less sleep stability with increasing age. The highest correlation with age was the increase in ambiguity of epochs scored as sleep (with a Pearson correlation coefficient of *r* = 0.62; *p* < 0.01) and the highest partial correlation with the arousal index (controlling for the effect of age) was the decrease in sleep continuity with increasing arousal index (*r* = −0.79; *p* < 0.01) (Anderer et al., 2022a).

Besides sleep duration, depth, and continuity, sleep restorative properties depend on the capacity of the brain to create periods of sustained stable sleep (Parrino et al., 2012). As discussed by Parrino et al. (2022), NREM sleep is bimodal with stable and instable periods, or alternatively conceptualized by the authors as effective and ineffective. Thus, the stability domain has only 2 forms of NREM sleep—stable and unstable where N3 is usually stable, N1 is always unstable, but N2 may be stable or unstable. To characterize the differences between apnea patients and healthy controls, we derived further features from the hypnodensity graph to cover these aspects of sleep physiology: the percentage of ambiguous NREM and REM epochs, where an epoch is defined as ambiguous if p(i)_MAX_ is ≤ 0.95; the percentage of stable NREM epochs, where two adjacent NREM epochs are considered as stable if (p(i)_N2_ + p(i)_N3_) > 0.95 and (p(i+1)_N2_ + p(i+1)_N3_) > 0.95; the percentage of stable REM epochs, where two adjacent REM epochs are considered as stable if p(i)_R_ > 0.95 and p(i+1)_R_ > 0.95; the percentage of NREM sleep depth defined as a weighted average of NREM probabilities: 100^*^((p(i)_N1_ + 2^*^p(i)_N2_ + 4^*^p(i)_N3_))/4). Table 5 provides the demographic data and the standard sleep parameters derived from the hypnogram for the controls and the apnea patients, as well as for the subgroup of mild to moderate apnea patients (apnea-hypopnea index, AHI <30) and severe apnea patients (AHI ≥ 30). The four groups did not differ in age and sex distribution. When compared to healthy controls the apnea patients did not differ in total sleep time, sleep efficiency, wake after sleep onset, sleep latency, REM latency and time in stage R, but showed increased time in N1 and decreased time in N2 sleep, while the time in N3 sleep was reduced only in patients with severe apnea.

**Table 5:** Demographic and standard sleep parameters for apnea patients and controls.

	**Controls**	**Apnea-All**	**Apnea-AHI <30**	**Apnea-AHI ≥ 30**
N	49	49	18	31
Age (years)	47.4 ± 15.4	50.8 ± 9.6	52.6 ± 8.4	49.8 ± 10.1
Sex (f/m)	7/42	7/42	4/14	3/28
AHI (#/hr TST)	1.4 ± 1.3	46.9 ± 28.7^*^	18.5 ± 7.4^*^	63.4 ± 22.8^*^
HB (%min/hr TST)	2.4 ± 3.9	187.6 ± 196.5^*^	49.4 ± 32.7^*^	267.9 ± 207.5^*^
ArI (#/hr TST)	15.1 ± 5.8	39.4 ± 23.1^*^	21.3 ± 9.1^*^	50.0 ± 22.2^*^
TST (min)	367.7 ± 54.9	382.0 ± 65.7	359.8 ± 84.3	394.8 ± 49.1
Sleep latency (min)	27.7 ± 23.5	25.6 ± 29.7	34.3 ± 44.5	20.5 ± 14.6
REM latency (min)	103.4 ± 44.2	199.9 ± 70.9	104.1 ± 47.3	127.2 ± 80.6
WASO (min)	80.8 ± 55.6	86.2 ± 55.4	102.4 ± 69.3	76.7 ± 44.0
Sleep efficiency (%)	77.4 ± 11.3	77.3 ± 12.8	72.2 ± 15.7	80.3 ± 10.0
N1 (min)	44.8 ± 22.3	121.7 ± 71.8^*^	70.6 ± 30.8^*^	151.3 ± 72.4^*^
N2 (min)	205.4 ± 42.7	158.8 ± 63.3^*^	169.4 ± 62.4^*^	152.7 ± 65.5^*^
N3 (min)	43.2 ± 30.4	29.4 ± 24.9	44.3 ± 22.5	20.7 ± 22.3^*^
R (min)	74.3 ± 24.6	72.1 ± 26.7	75.4 ± 29.5	70.1 ± 25.2
N1 (% TST)	12.5 ± 6.5	32.1 ± 18.0^*^	20.3 ± 9.3^*^	38.9 ± 18.4^*^
N2 (% TST)	55.8 ± 8.0	41.1 ± 13.5^*^	46.0 ± 9.7^*^	38.2 ± 14.7^*^
N3 (% TST)	11.7 ± 8.3	8.3 ± 7.5	13.4 ± 7.9	5.3 ± 5.6^*^
R (% TST)	20.0 ± 5.5	18.6 ± 6.4	20.3 ± 7.6	17.5 ± 5.4

* p <0.01 as compared to controls. AHI, apnea-hypopnea index; HB, hypoxic burden; ArI, arousal index; TST, total sleep time; WASO, wake after sleep onset.

**Figure 7:**
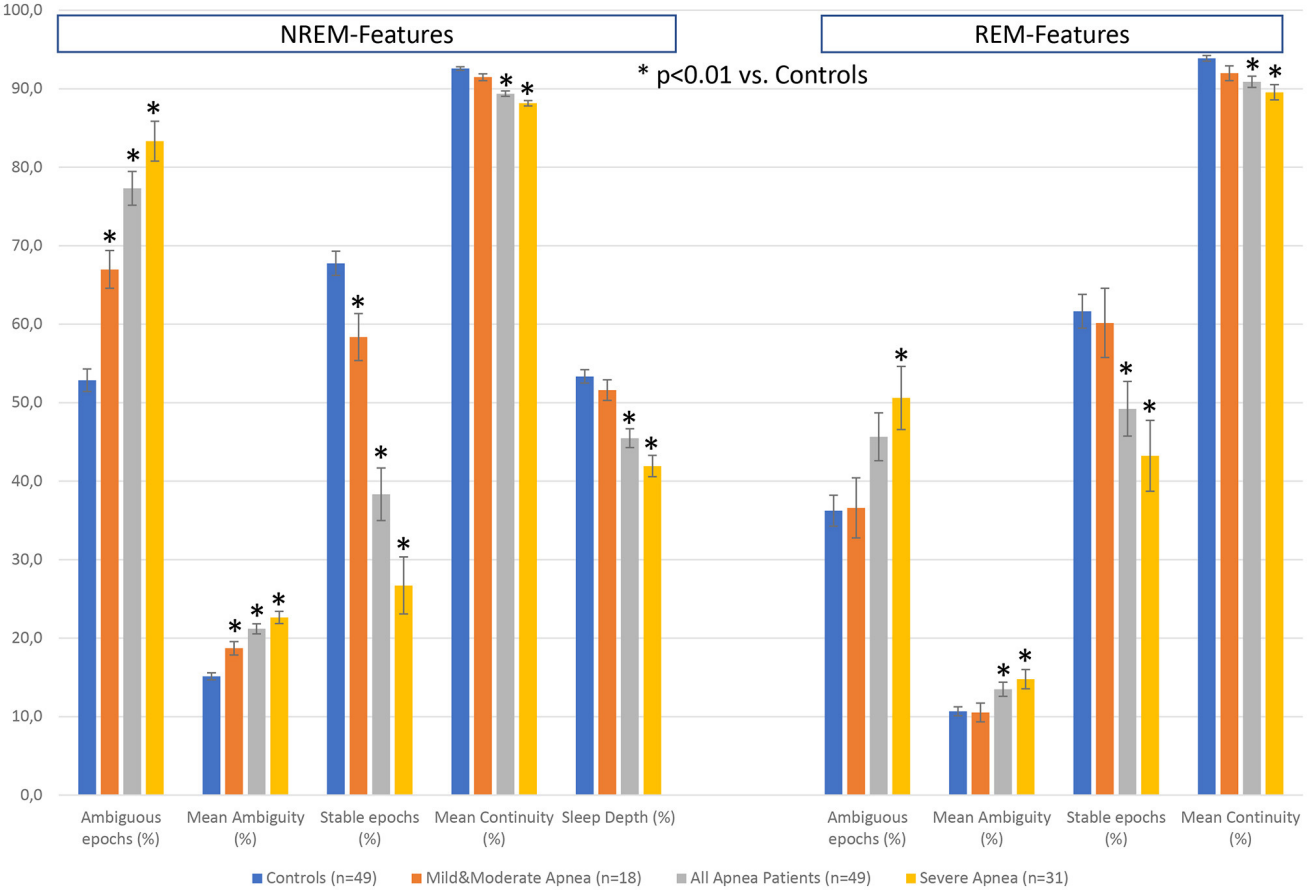
Differences in hypnodensity-derived features between patients with sleep apnea and age- and sex-matched healthy controls (*n* = 2 × 49). The left part depicts hypnodensity-derived NREM-features and the right part REM-features for the controls and the three patient groups mild & moderate apnea with AHI <30 (*n* = 18), all 49 apnea patients, and severe apnea with AHI≥30 (*n* = 31). *Indicates significant differences based on independent samples *t*-tests between patients and controls at *p* < 0.01.

Figure 7 summarizes the differences in the additional features between the 3 groups of apnea patients and age- and sex-matched healthy controls for the 5 NREM-features and the 4 REM-features derived from the hypnodensity graph. Concerning the differences between mild-to-moderate apnea and controls, we observed significant increases in the percentage of ambiguous NREM epochs (67 vs. 53%) and in the amount of the mean ambiguity (19 vs. 15%) as well as significant decreases in the percentage of stable NREM epochs (58 vs. 68%). These findings reflect the reduction of stable NREM sleep in patients with mild-to-moderate apnea (see also the significant shift from N2 to N1 sleep in Table 4). In contrast, REM sleep features are not significantly different between mild-to-moderate apnea and controls. While standard REM parameters such as REM latency and time and percentage in stage R are, even in patients with severe apnea, not significantly different to controls (Table 4), the hypnodensity-derived REM sleep features show significant increases in ambiguity and decreases in REM sleep stability in severe apnea (Figure 7). Parameters not determinable from the classical hypnogram, such as sleep stage ambiguity, reflecting the uncertainty of manual expert scorers, as well as sleep stage continuity, reflecting epoch-to-epoch changes of these uncertainties, may give valuable additional insights in the effects of different disorders in sleep architecture. A possible source of this ambiguity captured by the hypnodensity may be sleep stage shifts occurring within one 30-s epoch. Korkalainen et al. (2021a) used a deep learning approach based on the traditional 30-s epoch duration as well as based on shorter epoch durations (15-, 5-, 1-, and 0.5-s) to evaluate differences in sleep architecture between obstructive sleep apnea (OSA) severity groups. The authors reported decreases in sleep continuity with increases in OSA severity using Cox proportional hazards ratio or Kaplan–Meier survival curves, and these group differences became larger the shorter the epoch duration used was. The U-sleep model as presented by Perslev et al. (2021) can evaluate sleep architecture with even higher temporal resolution of up to 128 Hz which could provide additional diagnostic information and possible new ways of analyzing sleep. Interestingly, as shown recently by Fiorillo et al. (2023a) the U-sleep architecture successfully encoded sleep patterns even from non-recommended electrode derivations based on a large and heterogeneous dataset of 28,528 PSG recordings from various sleep centers (Fiorillo et al., 2023a). The authors wonder, given the criticisms of the AASM rules, the limited interrater reliability of manual scoring according to these rules, and the complexity of sleep, whether an unsupervised deep learning sleep scoring algorithm (i.e., without using manual sleep scorings as training targets) might be a better approach to describing human sleep.

Future studies in larger samples of patients with sleep related respiratory disturbance including measures of clinical outcome will be necessary to assess the relevance of hypnodensity-derived features for the development of physiological biomarkers. Of course, such endeavor should not be limited to apnea patients. Penzel et al. (2017) suggested that physiological biomarkers might be appropriate to characterize functional characteristics, as seen in the variety of sleep disorders. As stated above, the first promising examples for the construction of narcolepsy biomarkers including variables derived from hypnodensity have been already published (Stephansen et al., 2018; Cesari et al., 2022).

## Hypnodensity based on autoscoring using cardiorespiratory signals

HSAT studies are increasingly used as an alternative to PSG studies to diagnose SDB (Rosen et al., 2018). While less rich than the traditional PSG, HSATs are considerably less expensive due to a reduced cost of equipment and lower setup effort, enable increased access in remote or underserved areas, higher patient turnover, are much more comfortable and thus, less disruptive of sleep, and importantly, enable the opportunity to monitor patients in conditions that are more representative of their habitual sleep (Kim et al., 2015; Kundel and Shah, 2017; Rosen et al., 2018). HSAT studies record as a minimum set of signals, airflow, pulse oximetry, and respiratory effort for identification and classification of apneas and hypopneas. Relying on this reduced signal montage has obvious drawbacks compared to PSG studies. The absence of neurological signals required for manual sleep staging means that the AHI cannot be determined based on manual scoring. Instead, total sleep time is substituted by either monitoring time or recording time for calculation of the respiratory event index (REI) (Zhao et al., 2017). Reliance on the REI in place of the AHI results in reduced SDB diagnostic sensitivity that is not easily quantifiable, without knowing the amount of wakefulness in the individual recording (Bianchi and Goparaju, 2017). Further, there is no ability to screen for REM-related OSA or identify abnormalities in sleep architecture which may impact the subsequent treatment plan or signify the need for further testing (Kapur et al., 2017).

This limitation of HSATs motivated attempts to leverage the known expression of autonomic nervous system activity in sleep by analyzing cardiorespiratory signals, which are in fact routinely recorded with these polygraphic systems: the non-REM progression from N1 to N3 is typically accompanied by an increase in cardiovagal drive and parasympathetic activity, which translates to a lower heart rate, more regular breathing, and an increased respiratory mediation of heart rate variability (Eckert and Butler, 2016; Lanfranchi et al., 2016). REM sleep is characterized by a state of autonomic instability where sympathetic and parasympathetic nervous system activity fluctuate, producing abrupt changes in heart rate, and irregular breathing. In the absence of neurological signals, these algorithms are typically limited to the estimation of the stages wake, light sleep (LS; comprising the combination of N1 and N2), deep sleep (DS; corresponding to N3), and REM sleep. The differentiation between N1 and N2 based on neurological signals requires not only the timing of arousals but also sleep spindles and k-complexes, neither of which are available with cardiorespiratory inputs. These algorithms, of course, cannot mimic human scoring, since there are no rules, nor is it feasible, to visually relate changes in heart rate and respiration to sleep stages. However, advanced AI methods can often leverage, and go beyond what humans can possibly encode, to find relations in the data based on patterns that span entire recordings, while simultaneously analyzing numerous characteristics of the various signals.

**Figure 8:**
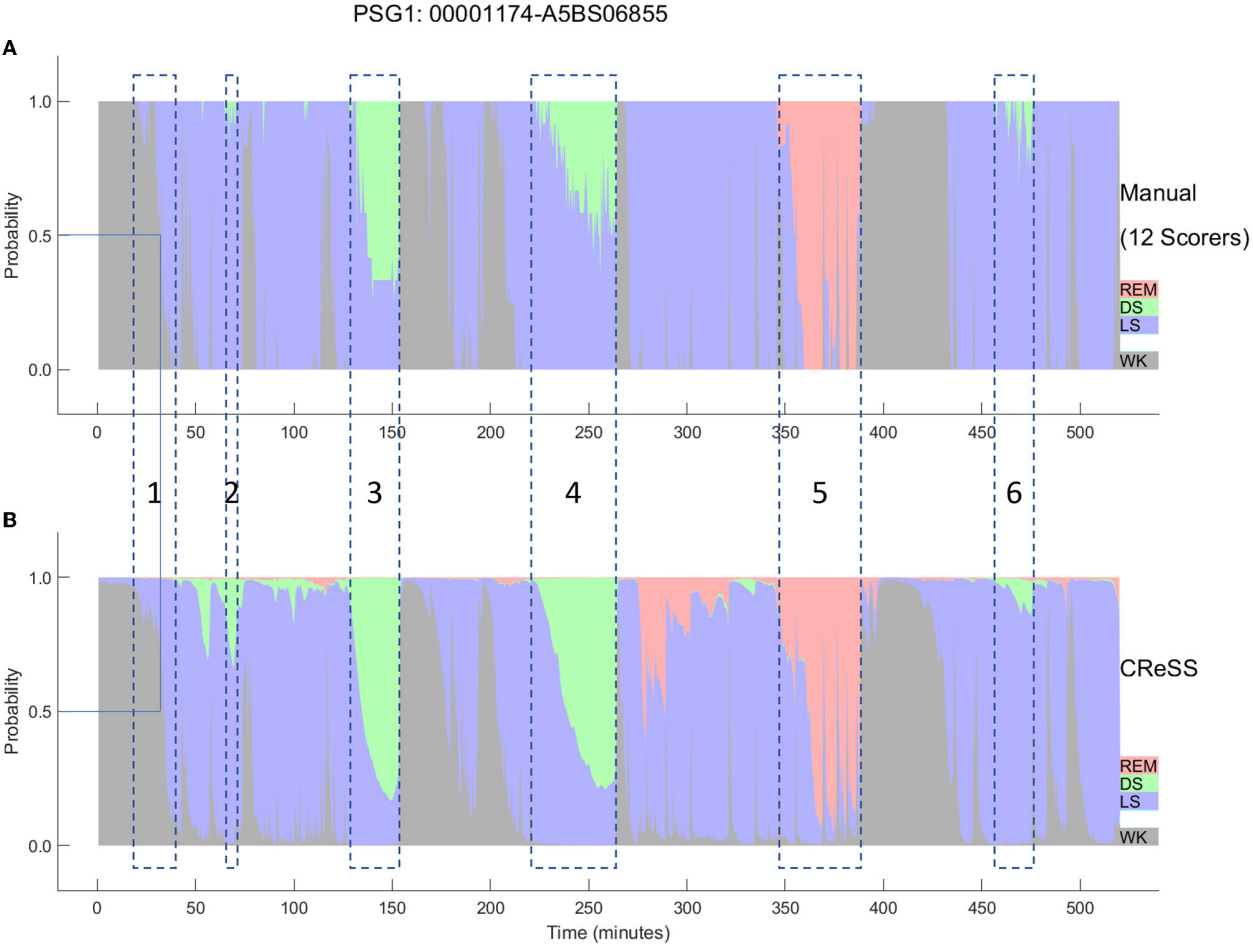
A representative example comparing the hypnodensities derived from 12 manual scorings **(A)** and from cardiorespiratory autoscoring **(B)** for the same study shown in Figure 2 (PSG1: OSAS patient, male, 76 years). The color codes are WK, gray; REM, red; LS, blue; DS, green. The time period depicted in box 1 highlights the sleep onset period with the actual sleep onset at W_prob_ <0.5 indicated as solid line; Boxes 2, 3, 4, and 6 indicate time periods where at least 2 scorers have scored N3; Box 5 indicate the time period where at least one scorer has scored R. Note the similarity of the manually-derived and the cardiorespiratory autoscoring-derived sleep stage probabilities.

**Figure 9:**
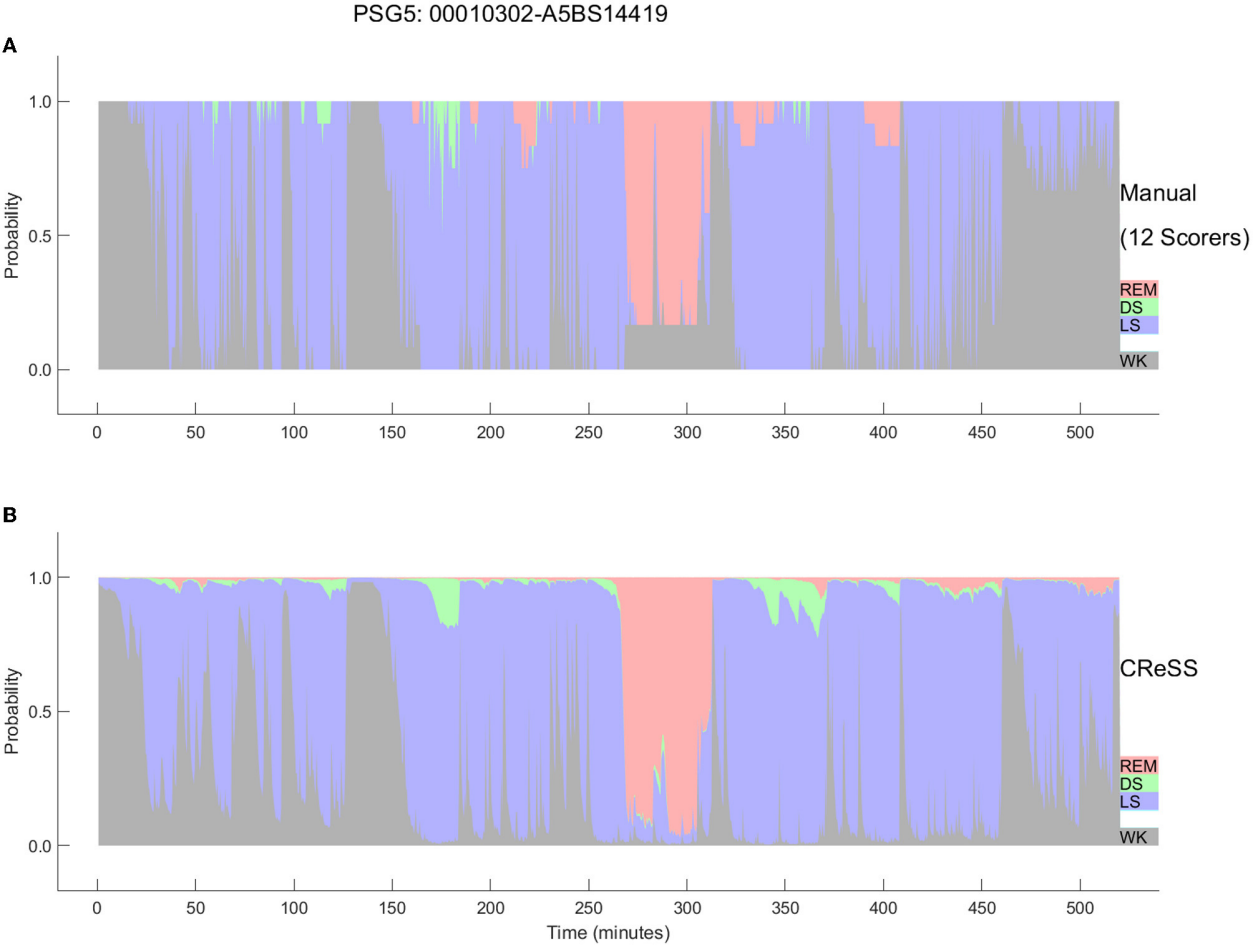
Comparison between the hypnodensities derived from 12 manual scorings **(A)** and from cardiorespiratory autoscoring **(B)** for the study with the worst agreement between manual scorers (i.e., the same study as in Figure 4, PSG5: Hypersomnia with sleep apnea, male, 78 years). The color codes are WK, gray; REM, red; LS, blue; N3, green. Note the similarity of the manually-derived and the cardiorespiratory autoscoring-derived sleep stage probabilities.

The AI-based Somnolyzer-CReSS algorithm uses as input cardiorespiratory signals, and outputs sleep stage probabilities per 30-s epoch for stages Wake, LS, DS and REM sleep (Bakker et al., 2021). The CReSS-derived probability curves can be directly compared to the probabilities derived from multiple manual scorings using neurological signals as input. Figures 8, 9 compare the manually-derived sleep stage probabilities to the sleep stage probability of the CReSS autoscoring for the two studies shown in Figures 5, 6. Note that the probabilities for N1 and N2 are summed up to a single LS probability. The ICCs for absolute agreement between the two probability curves are 0.91 for PSG 1 (Figure 8) and 0.84 for PSG 5 (Figure 9). While these correlation coefficients are slightly lower than the coefficients between autoscoring based on neurological signals and multiple manual scorings (0.97 for PSG1 and 0.89 for PSG 5), they still indicate good agreement between cardiorespiratory- and manually- determined sleep stage probabilities. In Figure 8, we highlighted the same 6 periods as in Figure 5. Box 1 comprises again sleep onset with increasing LS probability via sleep onset, which is the first epoch with sleep probability higher than wake probability (solid line), to definite sleep with LS probability > 0.95 at the end of the box 1. Boxes 2, 3, 4, and 6 indicate periods were at least two experts scored N3. Note that the DS probabilities derived from CReSS autoscoring closely resemble the N3 probabilities derived from the 12 manual scorings. Finally, also for the R probabilities (box 5) the manually- and autoscoring-based curves match in terms of timing and magnitude. Interestingly, even for the study with the worst agreement between scorers, the manually- and autoscoring-based probability curves match closely (Figure 9). The ICCs between manually-derived probabilities and CReSS-derived probabilities range from 0.81 to 0.95 (mean: 0.88 ± 0.05) for the 10 studies, indicating good agreement between the probability curves.

Consequently, the CReSS-derived hypnodensity also reflects a good estimate of the epoch-by-epoch ambiguity of manual scorings. The fact that very similar probabilities are derived from cardio-respiratory signals and neurological signals suggests that many of these uncertainties between sleep stages manifest in both, the central and the autonomic nervous system activity. This supports the view that states in-between two sleep stages are normal physiological states and that much of the uncertainty observed in sleep scorings is of an aleatoric nature, limiting the potential for further increases in inter-scorer agreement by efforts in improving scoring rules, training, or models. In a recently introduced framework to analyze uncertainty in sleep staging, van Gorp et al. (2022) differentiate aleatoric uncertainty, that arises from biological factors (such as age, drugs, pathologies, or local sleep) or measurement factors (such as placing of electrodes or interferences and artifacts), from epistemic uncertainty that arises from a lack of knowledge about the data or the optimal model.

## Agreement between sleep parameters derived from cardiorespiratory signals with sleep parameters derived from full PSG signals

Most of the early algorithms for cardiorespiratory sleep staging relied on manually engineered features carefully crafted to capture changes in autonomic nervous system activity during sleep, leveraging domain knowledge of sleep and cardiorespiratory physiology. In 2015, Fonseca et al. (2015) presented an algorithm to estimate sleep based on cardiorespiratory signals using manually-engineered features and a linear discriminant classifier, and reported a Cohen's kappa of 0.49 for the 4-stage comparison validated in 48 healthy subjects. By incorporating time information and replacing the classifier by conditional random fields, Cohen's kappa increased to 0.53 in 100 healthy subjects (Fonseca et al., 2018). In 2017, Tataraidze et al. (2017) reported a kappa of 0.56 based on respiratory inductance plethysmography (RIP) signals using an extreme gradient boosting classifier in 658 healthy subjects and Beattie et al. (2017) reported a kappa of 0.52 based on photoplethysmography (PPG) and actigraphy signals using a linear discriminant classifier in 60 healthy controls. In recent years, various machine learning approaches for scoring sleep based on cardiorespiratory signals have been developed and validated in internal and external datasets. Cohen's kappa for the 4-stage comparison (wake, light sleep, deep sleep, REM) were in the average 0.56 ± 0.12 for 11 datasets with internal testing (cross-validation or hold-out validation) (Li et al., 2018; Radha et al., 2019; Wei et al., 2019; Sridhar et al., 2020; Huttunen et al., 2021; Zhao and Sun, 2021; Garcia-Molina and Jiang, 2022) and 0.47 ± 0.15 for 10 datasets with external testing (Fonseca et al., 2020; Sridhar et al., 2020; Sun et al., 2020b; Bakker et al., 2021; Garcia-Molina and Jiang, 2022).

The Somnolyzer-CReSS algorithm was validated in a test set of 296 PSGs from the Multi-Ethnic Study of Atherosclerosis [MESA (Chen et al., 2015)] and achieved a kappa value of 0.68 and in a second test set of 296 PSGs from the Sleep Heart Health Study [SHHS (Quan et al., 1997; Redline et al., 1998)], a kappa value of 0.64, which are the two highest kappa values for external testing of cardiorespiratory sleep staging algorithms reported to date (Bakker et al., 2021). Sensitivity and precision for detecting wakefulness based on cardiorespiratory signals was 76.0 and 88.1%, respectively. This indicates good performance of the cardiorespiratory sleep staging for discriminating wake and sleep, which is important for determining total sleep time, and consequently, indices relating the number of respiratory events or the hypoxic burden (HB) to the hours of sleep. When compared to indices computed based on the duration of recording or monitoring time, the indices related to CReSS-determined total sleep time show a higher sensitivity, specifically in recordings with a significant amount of wake periods. To demonstrate the clinical relevance of CReSS, we determined the number of correctly diagnosed patients by HSAT as compared to the gold standard AHI in the 296 studies from the MESA dataset for a threshold of 15 events per hour. Using the CReSS-derived TST instead of the recording time as denominator for the calculation of the indices reduced the false negative diagnosis from 33 patients (11.1%) to only 5 patients (1.7%). Moreover, sensitivity and precision for detecting REM sleep based on cardiorespiratory signals was 85.3 and 79.6%, respectively (Anderer et al., 2022b). This indicates the good performance of CReSS for discriminating REM sleep from Wake and NREM sleep. Using the definition for REM-related OSA by Mokhlesi and Punjabi (2012) (i.e., an AHI_NREM_ of fewer than 5 events/h and an AHI_REM_ of at least 5 events/h with at least 30 min of REM sleep), we achieved a sensitivity of 91% and a specificity of 98% for detecting REM-related OSA by means of CReSS as compared to gold standard PSG scoring. This suggests that REM-related OSA can be detected based on CReSS-determined REM sleep with a clinically acceptable accuracy (Anderer et al., 2022b).

**Figure 10:**
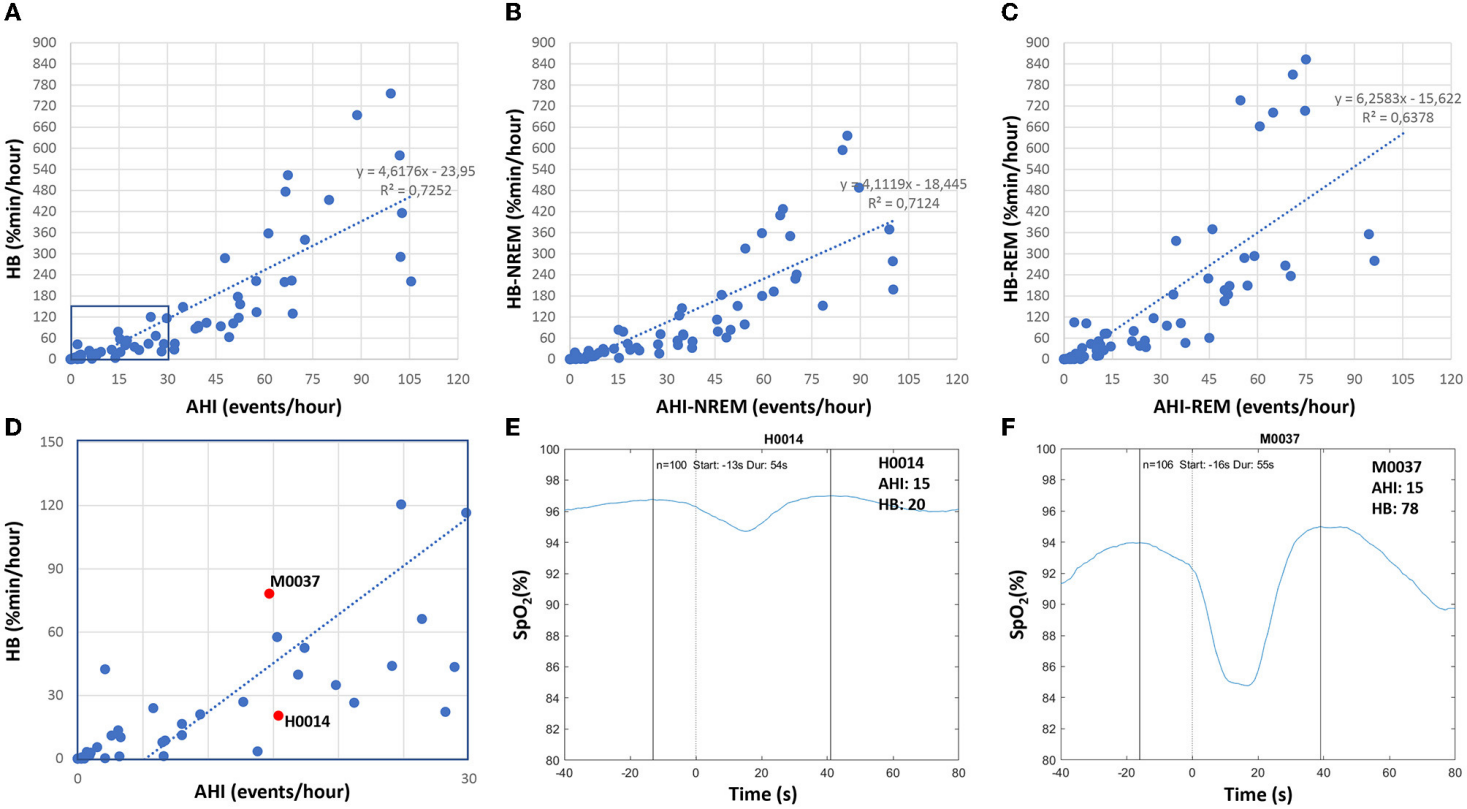
Correlation between apnea-hypopnea index (AHI) and hypoxic burden (HB) in patients with sleep disturbance (*n* = 80; 49 apnea patients, 26 insomnia patients, 5 PLMD patients). Upper part: scatter plots relating the AHI to the HB for TST **(A)** as well as for NREM sleep **(B)** and REM sleep **(C)**. Lower part: enlarged scatterplot of AHI vs. HB with red marks for two studies with approximately the same AHI of 15, but with significantly different HB values **(D–F)** show the averaged oxygen saturation curves, time-aligned by the termination of the respiratory events (time = 0), which are used to determine the subject-specific search window for the two studies marked in red in **(D)**.

In addition to the AHI, which indicates the number of respiratory events per hour of sleep, we determined the hypoxic burden as proposed by Azarbarzin et al. (2019). The hypoxic burden is determined by measuring the respiratory event-associated area under the desaturation curve from pre-event baseline. The authors showed, in a large sample from the Sleep Disorder in Older Men study [MrOS (Orwoll et al., 2005)] and the SHHS (Quan et al., 1997; Redline et al., 1998), that the hypoxic burden strongly predicted cardiovascular disease-related mortality, indicating that not only the frequency (as measured by the AHI), but the depth and duration of the desaturations caused by sleep-related upper airway obstructions (as measured by the hypoxic burden), are important disease-characterizing features. Figure 10 shows based on Somnolyzer autoscoring, in the upper part, scatter plots relating the AHI to the HB for TST as well as for NREM and REM sleep. As can be seen, the HB for events occurring during REM sleep is, in our dataset, ~50% larger than for events in NREM sleep. Note that patients with a relatively low overall AHI may be experiencing severe OSA during REM, which is particularly important given that events taking place during REM are longer, and are associated with more pronounced hypoxemia, higher sympathetic activation, and greater surges in blood pressure and heart rate (Findley et al., 1985; Peppard et al., 2009; Lechat et al., 2022a,b). This characteristic of the disease may very well help explain the link between REM-related OSA and its association with adverse cardiovascular, metabolic, and neurocognitive outcomes (Varga and Mokhlesi, 2019).

In their comprehensive review on the hypoxic burden in obstructive sleep apnea , Martinez-Garcia et al. (2023) suggested a threshold HB > 60% min/h (i.e., 15 min of 4% desaturation every hour) to identify patients who are at increased risk of cardiovascular morbidity and mortality. In the lower part of Figure 10, we enlarged a portion of the scatterplot of AHI vs. HB and marked the values for two studies with approximately the same AHI close to 15, but with very different HB values (20.4% min/h and 78.2% min/h). In addition, we show for both studies the averaged oxygen saturation curves, time-aligned by the termination of the respiratory events, which are used to determine the subject-specific search window. While the number of respiratory events (100 and 106) and the duration of the search window (54s and 55s) are almost identical, the averaged desaturation is much deeper in subject M0037, reflecting the large difference between the two studies.

**Figure 11:**
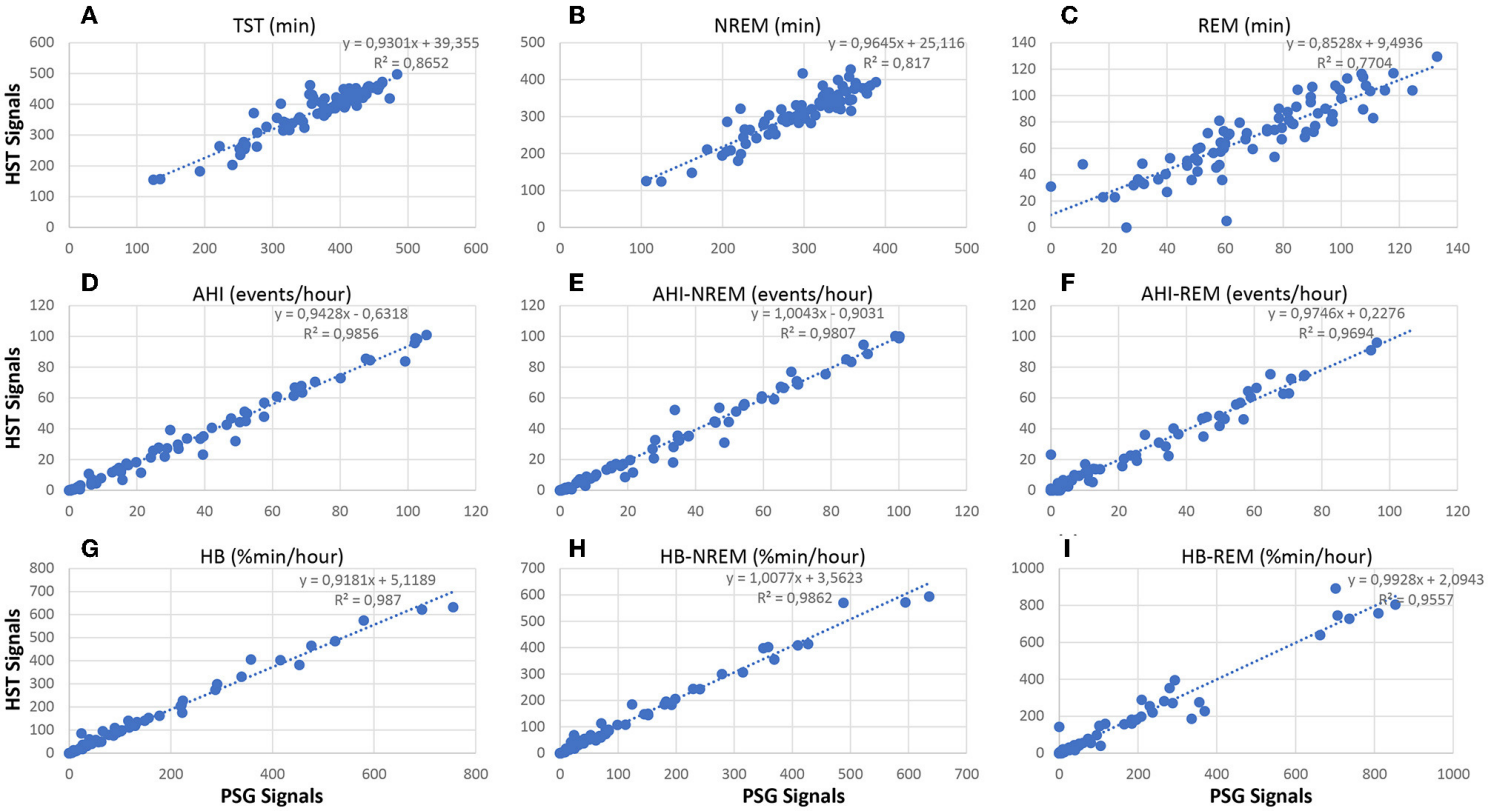
Correlation between neurological signal-based and cardiorespiratory signal-based sleep parameters in patients with sleep disturbance (*n* = 80; 49 apnea patients, 26 insomnia patients, 5 PLMD patients). Upper part: scatter plots relating TST **(A)**, time in NREM **(B)** and time in REM **(C)** based on the analysis using PSG signals with the values obtained from the analysis using HSAT signals only. Middle part: scatter plots relating AHI **(D)**, AHI in NREM sleep **(E)** and AHI in REM sleep **(F)** based on the analysis using PSG signals with the values obtained from the analysis using HSAT signals only. Lower part: scatter plots relating HB **(G)**, HB in NREM sleep **(H)** and HB in REM sleep **(I)** based on the analysis using PSG signals with the values obtained from the analysis using HSAT signals only.

Figure 11 compares standard sleep parameters based on Somnolyzer autoscoring derived from full PSG signals vs. the same parameters derived from HSAT signals in patients with sleep disturbance from the SIESTA database. Hypopneas were scored in the PSG studies using the 3% oxygen desaturation and/or arousal rule, and in the HSAT studies using 3% desaturation and/or autonomic response (heart rate increase ≥ 5bpm) to enable a direct comparison between the PSG- and the HSAT-derived indices. In addition to the 49 apnea patients, we also included the 26 patients with insomnia related to generalized anxiety disorder or depression, and the 5 patients with periodic limb movement disorder in the analysis to cover the full spectrum from no to severe SDB. In addition to the scatter plots for TST, AHI and HB per hour total sleep time, the scatter plots for NREM and REM are shown. The CReSS algorithm estimated TST as well as time in NREM and REM sleep with high accuracy. The ICC for absolute agreement are for TST 0.92 (95%–CI 0.83 to 0.95) for time in NREM 0.88 (95%–CI 0.75 to 0.93), and for time in REM 0.88 (95%–CI 0.82 to 0.92). Consequently, the indices per hour sleep also show almost perfect agreement between the analysis based on PSG signals and the analysis based on the reduced HSAT montage (all ICCs ≥ 0.98 with a 95%–CI from 0.97 to 0.99). Thus, analyses based on signals recorded typically in HSAT by means of CReSS are a valid alternative to full PSG studies in patients with suspected OSA for determining the severity based on the AHI and HB per hour sleep and for diagnosing REM-related OSA.

## Conclusions and future directions

There is convincing evidence that manual sleep staging, even when performed by experienced, well trained, and motivated scorers without the usual time constraints of clinical routine, results in significant interrater differences. We have shown in three independent datasets scored by six to twelve experienced scorers that sleep stage ambiguity is the rule rather than the exception (Bakker et al., 2023). Recent papers investigating reasons for this ambiguity discuss scorers' uncertainty in applying the rules as well as contradictory patterns within one epoch as possible explanations (van Gorp et al., 2022; Huijben et al., 2023). van Gorp et al. (2022) introduced a theoretical framework to analyze uncertainty in sleep staging, differentiating aleatoric uncertainty that arises from biological factors (such as age, drugs, pathologies, or local sleep) or measurement factors (such as placing of electrodes or interferences and artifacts) and epistemic uncertainty that arises from a lack of knowledge about the data or the optimal model. In standard sleep staging, scorers are forced to decide based on the information obtained in the EEG, EOG, and chin EMG signals. This process involves matching the pattern observed in an epoch with a template or prototype and putting them into the context with patterns from previous epochs. Depending on the scorers' personal template, this may result in significantly different sleep parameters derived from the manually-scored hypnogram as shown in Table 1 for one PSG. In autoscoring systems these different interpretations can be modeled by varying the sensitivity settings for the detection of sleep/wake related features such as sleep spindles, k-complexes, slow waves or arousals without changing the scoring rules. This provides evidence that large parts of the inter-scorer differences in the derived sleep parameters are not due to violations of the scoring rules by one or the other scorer, but rather due to the room for interpretation left open by the visual identification of these sleep/wake related patterns. These interpretations range from high sensitivity to high precision sometimes resulting in extreme differences where one expert scores 67 min of N3 (high sensitivity for slow wave detection) while another expert scores no N3 sleep at all (high precision in slow wave detection) in the same study, despite following the same rule (≥20% of the epoch consisting of slow wave activity). This means that a decision for an epoch does not only affect this one epoch but can have consequences for a whole series of subsequent epochs resulting in the observed large differences in sleep parameters.

When we compared sleep parameters averaged over 10 PSGs between all 66 possible pairs of 12 scorers, we found in 61 of these pairs a significant *t*-value at *p* < 0.01 in at least one of 5 tested parameters (TST, time in N1, N2, N3, and R), most frequently in the time spent in N3 sleep. This implies that when comparing two conditions (patients vs. controls, baseline vs. therapy, etc.) that were scored by different (groups of) scorers, it cannot be distinguished whether significant results describe a difference between the conditions or a bias of the scorers. Possible solutions to this problem include having all PSGs from a study (at least all PSGs from one subject, in case of repeated measurements) scored by the same expert, having all PSGs scored by multiple manual scorers, or by using a clinically validated autoscoring algorithm.

In future research, it is therefore strongly recommended that the performance of sleep scoring algorithms should be independently validated in datasets which were completely unseen by the models both during training and internal validation, that are representative of the population to be tested, and ideally, that are collected in different centers and scored by different (pools of) human experts. In fact, the AASM has announced such an AI/Autoscoring Pilot Certification program at their website. The program intends to test the various scoring solutions to one and the same external dataset with representative recordings vs. multiple manual expert scorers. This will allow a direct comparison of the performance of the published algorithms and will give the sleep centers an objective measure for deciding which algorithm to use. Since multiple human expert scorings will be available in this project, the hypnodensity graphs provided by the different algorithms could be compared to the hypnodensity graph based on human scorings so that the hypnodensity from certified algorithms may be established as standard representation of sleep into the clinic. In this context, it will be an interesting topic of future research to determine and establish well-accepted metrics for assessing the quality of hypnodensity graphs. While the overall agreement on the traditional five-stage hypnogram is often measured using the Cohen's kappa coefficient, the F1 score or class-wise metrics like the Mathews correlation coefficient, no metric for comparing sleep stage probabilities has been widely adopted by the field of sleep medicine, yet. Possible metrics include the ICC to compare probabilities for individual stages, their average (macro average), their average weighted by the sum of the probabilities per stage (weighted macro average) or the ICC based on the concatenated probability curves over all five stages (micro average), as well as the ACS to compare sleep stage probability distributions (Bakker et al., 2023; Fiorillo et al., 2023b). In fact, both metrics yield very similar results and others such as cross-entropy or Kullback–Leibler divergence might become relevant for measuring the difference between the sleep stage probability distributions based on multiple manual scorings and autoscoring.

We presented examples of potential valuable hypnodensity-derived features such as sleep stage ambiguity, continuity, depth, and stability for describing differences between patients with sleep apnea and healthy controls. Stephansen et al. (2018) and Cesari et al. (2022) derived up to 1000 features of sleep structure, transitions, and instability from the hypnodensity to train a classifier for diagnosing narcoleptic patients. Further examples for hypnodensity-derived features including pre-softmax features as well as features obtained from unsupervised learning are also being researched (Huijben et al., 2023). Future research should evaluate and test these features for their usefulness in biomarker research.

Concerning HSATs, AI-based cardiorespiratory sleep staging offers reliable estimates of total sleep time, as well as time spent in light, deep, and REM sleep (Li et al., 2018; Radha et al., 2019; Wei et al., 2019; Sridhar et al., 2020; Sun et al., 2020b; Bakker et al., 2021; Huttunen et al., 2021; Zhao and Sun, 2021; Garcia-Molina and Jiang, 2022; Pini et al., 2022). This allows for determining indices of SDB severity per hour of sleep as well as per hour of NREM and REM. In contrast with the classical recommendations for HSATs which do not measure sleep but instead rely on the total monitoring/recording time, the accurate estimates of sleep time can be used to increase the sensitivity of these tests, making the indices immune to the duration of wakefulness in these unsupervised studies. In addition, they allow the identification of patients with REM-related obstructive sleep apnea, the computation of hypoxic burden as a function of the total sleep time as well as the times in NREM and REM.

With the recent advances in autoscoring in general, and the development of hypnodensity in particular, it is increasingly clear that AI may have a defining role in future sleep research, and eventual clinical applications. The development of new biomarkers may help us understand pathophysiological mechanisms that were until now simply not accessible from hypnograms scored by individual human experts. On the other hand, this technology shows promise in the routine home testing and diagnosis of SDB. By enabling an estimate of TST with HSATs, AHI and HB across total sleep time and during REM can be estimated, until now an exclusive of the more inconvenient and expensive PSG studies. To further improve the estimation of these indices, several attempts to determine autonomic arousals as surrogate of cortical arousals for the confirmation of hypopneas have been published (Pillar et al., 2002; Olsen et al., 2018; Li et al., 2020). Taranto-Montemurro et al. (2023) recently reviewed challenges and progress in the development of a combination of noradrenergic and antimuscarinic drugs for the treatment of OSA. The authors concluded that there are still hurdles in quantifying presence and severity of OSA to fully understand the impact of treatment. The authors concluded that the usage of alternative measures to the standard AHI, such as the HB might better represent treatment effects on the ventilatory deficit associated with upper airway obstruction. In a recent review, Korkalainen et al. (2021b) discussed self-applied home sleep recordings including wearable sensing solutions and AI-based scoring for screening and long-term monitoring of sleep disorders. Besides the obvious advantages in clinical practice, the larger scale and higher throughput of AI-enabled HSATs may also facilitate larger population-wide research studies that help us understand the link between SDB and other health conditions and outcomes.

## Author contributions

PA, MR, and AC contributed to conception and design of the presented topic review and drafted the manuscript. RV, ES, and PF contributed to the final version of the manuscript. All authors contributed to manuscript revision, read, and approved the submitted version.
